# Cognitive Ability and Non-Ability Trait Predictors of Academic Achievement: A Four-Year Longitudinal Study

**DOI:** 10.3390/jintelligence13070079

**Published:** 2025-06-30

**Authors:** Phillip L. Ackerman, Ruth Kanfer

**Affiliations:** School of Psychology, Georgia Institute of Technology, Atlanta, GA 30332, USA; rkanfer@gatech.edu

**Keywords:** achievement, cognitive ability, personality, interests, validity

## Abstract

Prediction of individual differences in academic achievement is one of the most prominent longstanding goals of differential psychology. Historically, the main source of prediction has been measures of intelligence and related cognitive abilities. Researchers have suggested that non-ability traits, such as personality, may also provide useful information in predicting academic achievement. Meta-analyses have indicated that there are significant correlations between such variables, but most of the existing studies have been conducted with cross-sectional designs, or with a limited inclusion of intelligence/cognitive ability variables, making it difficult to determine whether the non-ability measures provide incremental predictive validity for academic achievement. In this longitudinal study, both extensive cognitive ability and non-ability trait measures (personality, interests, self-concept/self-estimates of abilities, and motivational traits) were administered at the beginning of secondary school, and criterion measures of ability and academic achievement were obtained after four years of secondary school. The results indicate that although non-ability trait measures have significant and meaningful correlations with the criterion measures, their incremental predictive validity over cognitive abilities alone is somewhat diminished. Nonetheless, there is potential utility for including assessments of non-ability traits for predicting future academic performance and elective course enrollments.

## 1. Introduction

In the United States and some other countries, secondary education (typically 9th–12th grade in the US) represents a partial transition from a common curriculum where students are expected to all complete the same courses (primary school), to one where the students are able to select at least some courses (as electives), in domains such as languages, science, arts and humanities, and so on. When *choice* is introduced into the educational system at this point, there is potentially a greater role for non-ability influences to determine the direction of the students’ effort in achieving educational goals. That is, from secondary education onward, an individual’s personality, interests, motivation, and self-concept may play a larger role in academic achievement, when compared to their performance with compulsory curricula, which may be more influenced by cognitive abilities and less by preferences and interests (e.g., see Hessen and Kuncel 2022). By the time students reach decisions to pursue particular career paths or post-secondary educational opportunities, the cognitive ability and non-ability influences (especially vocational interests) clearly are influential, but the indicators of such influence are perhaps better reflected as choices, rather than continuous variables such as academic performance. Certainly the diminished role that cognitive/intellectual abilities have in predicting college/university performance, compared to the prediction of primary school performance is consistent with that interpretation (e.g., see Anastasi and Urbina 1997).

The current investigation is concerned with cognitive ability and non-ability predictions of indicators of academic achievement in a four-year longitudinal study of secondary school students (9th–12th grade). We administered predictive measures at the beginning of ninth grade, followed-up with ability and knowledge tests in 12th grade, and obtained transcripts of grades and elective course participation across the high school years. In particular, we were interested in the incremental predictive validity of non-ability predictors for academic achievement, when a broad battery of cognitive ability predictors were first entered into the prediction equation.

### 1.1. Prediction of Academic Achievement

The most prominent historical theme of intelligence assessment, mostly identified with Binet and Simon (1905/1973), proceeding through Terman (1916), Thurstone and Thurstone (1941), and numerous others, is tightly linked to the prediction of academic achievement, at least for applications for children and young adolescents. Indeed, this has been the main reason for creating intelligence tests to begin with. Therefore, it comes as no surprise that historical and ongoing research has shown that measures of cognitive ability are highly predictive of academic achievement indicators, especially measures of school grades, along with measures of academic aptitude and broad achievement (e.g., see Humphreys 1973; Roth et al. 2015). The history of intelligence and ability assessments is beyond the scope of the current paper, but see, for example, Anastasi and Urbina (1997); Carroll (1982); Thorndike and Lohman (1990) for reviews.

Most non-ability trait measures (e.g., personality, motivation), in contrast to intelligence tests, were not designed to predict academic achievement—their validation mainly depends on alignment with theory or in some cases, prediction of psychopathology (e.g., see Cronbach 1949). Historically, however, there are some non-ability trait measures that are purported to align with cognitive abilities (most notably, the personality traits related to ‘engagement’, such as Need for Achievement, Openness to Experience, Typical Intellectual Engagement; Ackerman and Heggestad 1997; von Stumm and Ackerman 2013), and motivational measures of Desire to Learn and Mastery (e.g., see Heggestad and Kanfer 2000). The linkage between these traits and cognitive abilities might imply positive associations with measures of academic achievement, but it is not clear what portions of such associations are due to unique aspects of these non-ability traits or due to their overlap with cognitive ability measures (Ackerman 1997).

In one meta-analysis of Five-Factor Model scales, Poropat (2009) found that for secondary education, only Conscientiousness (*r* = 0.19) and Openness (*r* = 0.10) accounted for more than 1% of the variance in measures of academic achievement. In a more recent meta-analysis, Mammadov (2021) reported that both Openness (estimated true score correlation = 0.22) and Conscientiousness (estimated true score correlation = 0.27) were substantially related to academic achievement in secondary school student populations. In addition, when considered from a perspective of incremental predictive validity, personality trait measures added 10.2% of the variance accounted for in the prediction of academic performance, beyond the 17.6% of the variance accounted for by cognitive abilities, yielding a total variance accounted for of 27.8%.

One specific study (Meyer et al. 2019) also provides insights into the influence of personality traits in predicting secondary school academic achievement across different domains, in the context of cognitive ability measures. Although this was a cross-sectional study with a sample of 3637 students, the authors found that Conscientiousness added incremental predictive validity for grades in mathematics, but not English, while Openness had incremental predictive validity for English, but not for mathematics, consistent with earlier research suggesting that openness-type trait measures are more strongly associated with measures of crystallized intelligence (Gc) than with fluid intelligence (Gf), and that English/domain-knowledge measures are more highly associated with Gc, while math achievement is relatively more strongly associated with Gf (e.g., see Ackerman and Goff 1994; Goff and Ackerman 1992).

Other empirical investigations and meta-analyses have pointed to Conscientiousness and Openness as the most robust predictors of individual differences in academic achievement in terms of grade point averages (e.g., see Brandt et al. 2019; Spengler et al. 2013).

Some modern interest assessments do have roots in predicting academic achievement, most notably Holland’s (1959) early work on predicting college/university performance from assessments of intellectual (investigative) and aesthetic (artistic) interests. Similar to the engagement traits, though, it is not clear whether any interest theme correlations with academic achievement are independent of the contributions of individual differences in cognitive abilities.

Finally, measures of self-concept, especially in the academic domain (e.g., see Shavelson et al. 1976) and self-estimates of abilities (e.g., see Ackerman and Wolman 2007) represent an individual’s own estimates of their cognitive abilities, which likely include both knowledge of previous academic experiences, and to some degree, one’s self-efficacy for performance on academic tasks (e.g., see Ackerman et al. 2013). As such, these measures may provide incremental predictive validity for future academic performance, especially when the individual has control over whether the individual seeks greater or lesser academic challenges, such as enrolling in more difficult or advanced courses that are electives rather than required courses.

#### 1.1.1. The Current Study

The current study was designed to investigate the patterns of elective college-level courses (Advanced Placement [AP]) taken during secondary school and the effects of completion of these courses on grades, abilities, and domain knowledge, as completed by the students across the 9th–12th-grade high school years (Ackerman and Kanfer 2012). The prediction of these criteria was inspired by Ackerman’s Intelligence-as-Process, Personality, Interest and Intelligence-as-Knowledge (PPIK) theory, and the construct of “trait complexes” (Ackerman and Heggestad 1997). Together, these frameworks propose that intellectual development is determined by the investment of cognitive and key non-ability variables, which are themselves organized into larger constellations (e.g., science/math, intellectual/cultural, social, and conventional) of positively related traits. The underlying PPIK theory proposes that academic and occupational criteria can be well predicted from a relatively small set of trait complex measures that include cognitive and non-ability families of constructs. This is a useful framework for applications because, for example, a guidance counselor would only have to integrate a small number of predictor measures for providing recommendations to students and other stakeholders in planning future course enrollments. Such an approach does not directly answer the question of what the individual trait-family constructs contribute to the overall prediction of individual differences in academic achievement. However, the collection of ability, personality, interest, self-concept/self-estimates of abilities, and motivational trait measures provides the basis for an investigation of the cognitive ability and non-ability contributions to individual differences in school achievement (defined as grade point average [GPA]), ability, and domain-knowledge criteria.

The principal advantage of the current study design is that we were able to simultaneously administer measures across a wide range of cognitive ability and non-ability constructs. Other studies have examined subsets of these measures or somewhat limited measures (e.g., two-items/dimension, see Spengler et al. 2013). However, in order to provide a thorough account of the independent and incremental validity of cognitive ability and non-ability constructs in predicting academic achievement measures, it is critical that there be a reasonably comprehensive sampling of the various constructs. Without broad sampling, comparative and incremental validity estimates may be somewhat misleading because of reduced reliability of the instruments, lack of suitable aggregation, and most importantly, the omission of relevant constructs. For example, a comparison of personality measures with a single estimate of ability, such as Raven’s Progressive Matrices test, would overlook the influence of verbal and mathematical abilities. A comparison of personality and ability measures only in predicting academic achievement might overlook the potential interactions between objective abilities, personality traits, and self-estimates of abilities in determining whether the students orient toward more or less challenging curricula, and thus influencing the overall academic achievement in a complex fashion.

No studies in the literature have attempted to measure such a diverse and extensive set of measures at the same time, including a longitudinal follow-up of academic achievement across four years of secondary education. The key question to be addressed in this paper is the individual and incremental validity of cognitive, affective, and conative trait measures administered in ninth grade to predict these 12th-grade criteria. In addition, because of the inclusion of data on elective course enrollment patterns, this study allows for additional investigation of the role of cognitive and non-ability variables on the students’ decision to enroll in challenging college-level courses during high school.

#### 1.1.2. Advanced Placement Course Completion

Over the past several decades, the U.S. high school educational system has provided students with a transition from a nearly universal core curriculum of required courses (most notably in the K-6 system), where students have little or no choice in elective courses, to the post-secondary educational system, with a vast array of college/university majors, specific vocational programs, and elective courses, among which students have a plethora of choices. Individual states mandate high school core curricula (e.g., specific numbers of courses in English, math, physical sciences, social sciences, and foreign languages). However, as students transition from 9th to 12th grade, they are often faced with opportunities to select a variety of elective courses, either as choices among different courses within a required curriculum (e.g., different selections for social sciences courses), or as choices outside the required curriculum (e.g., art, music, physics, calculus, computer science, psychology). In addition, the Advanced Placement (AP) program, which started in the 1950s as a means for talented high school students to complete college-level courses while in high school, has blossomed from an original set of 10 exams in core areas of study (e.g., “English composition, literature, Latin, French, German, Spanish, mathematics, biology, chemistry, and physics” (DiYanni 2006)) to 33 exams that span the original areas and diverse domains such as art history, environmental science, human geography, and macroeconomics. There has been an explosive growth in the number of AP exams administered, from about 10,000 in 1960 to a half-million exams in 1990, 1.5 million exams in 2002 (DiYanni 2006) to 3.2 million exams in 2010, completed by 1.8 million students (College Board 2011). Although AP courses are considered to be “elective” courses, they are seen by many stakeholders (namely teachers, students, counselors, college admissions personnel, and parents) to be an essential ingredient, if not a virtual requirement, for students to be accepted by selective colleges and universities. Many high schools have reacted to the AP program by devoting considerable resources to offering numerous AP courses and making AP courses available to interested and talented students. Students have benefitted from these programs by successfully completing AP exams, and in many cases, receiving college course credits on admission to college/university. These credits, in turn, are associated with both higher grades in college/university study and faster time-to-degree achievement.

To date, there has been a lack of significant progress in the educational research and application domains on how to best match students and elective courses of study at the high-school level in order to maximize outcomes. Teachers, counselors, students, and parents have relatively little in the way of integrated, research-based tools to make placement decisions. Decisions about course placement are variously based on grade cut-offs, teacher recommendations, subjective assessments of student interests, and similar considerations that are often difficult to integrate and may not lead to optimal success rates or optimal matches of student interests and other characteristics to course content choices. Traditional interest measures are only useful to the degree that interests capture a substantial degree of success variance, but that is not what they were designed for. Traditional cognitive tests are also problematic in isolation because they do not adequately sample many different domains in which courses are offered, and they do not assess the range of domain knowledge that is relevant to evaluating the individual’s prior experience and the results of specific interests. Finally, other potentially important traits (e.g., affect, motivation, self-concept) are typically not measured in any organized fashion in this population.

### 1.2. Research Questions

Based on prior investigations of individual trait-family relationships to academic achievement, we hypothesized that cognitive abilities would have the highest validities for predicting individual differences in future academic achievement. We also anticipated significant relationships between various measures of personality, self-concept, interests, and motivational traits on indicators of academic achievement (and the pattern of enrollment in AP courses). However, given a robust set of cognitive ability predictor variables, it was unclear whether these non-ability measures would provide significant and meaningful incremental predictive validity for individual differences in academic achievement. Thus, we did not make specific predictions for the degree of incremental variance accounted for by these variables. Given the historical basis for the dominance of cognitive ability measures for predicting academic achievement (especially, based on the fact that such measures are specifically designed to predict academic achievement, whereas the other measures are not constructed for such purposes), incremental validity was measured for non-ability measures *after* abilities were first entered into multiple regression equations.

Deciding which ability predictor measures to include in this kind of study is relatively straightforward, given a century or so of research and applications in this area. The consensus approach is to sample the main content domains of abilities (verbal, numerical, figural/spatial), which in turn can be aggregated into a general ability composite if desired. Deciding which non-ability variables to include in a study of this type is a difficult process, given the literally thousands of different non-ability constructs that have been developed. We were guided in this selection by previous research that specifically focused on both the broader construct relations between personality traits, vocational interests, and motivational traits (e.g., Ackerman 1997; Ackerman and Heggestad 1997) and the role of self-concept and self-estimates of abilities in predicting individual differences in academic performance (e.g., Ackerman et al. 1995). There are various theoretical accounts relating to each of these traits in determining differences in academic performance (e.g., for self-concept, see Marsh et al. 2018; Shavelson et al. 1976). However, we were agnostic about the ‘mechanisms’ by which these variables influence academic performance, partly because there is a high degree of item promiscuity among these various non-ability construct assessments (i.e., the same or similar items appearing under different non-ability construct measurements), and it is unclear which underlying trait identifiers are ultimately causal influences on academic performance.

The design of this over-arching study (Ackerman and Kanfer 2012) was to investigate the role of ‘trait complexes’ in predicting academic success of secondary school students. Trait complexes (see Ackerman and Heggestad 1997; Snow 1989) [“aptitude complexes”]) are constellations of related traits (across cognitive ability and non-ability constructs, such as personality, interests, motivation, self-concept and self-estimates of abilities) that represent shared variance and are hypothesized (e.g., Ackerman et al. 2013) to have synergistic appetitive (e.g., science/math and intellectual/cultural trait complexes) and aversive (e.g., clerical/conventional and social/enterprising) relations to academic orientation and academic achievement. From an applied educational perspective, one principal advantage of the trait complex approach is that even with the loss of some precision that accompanies the reduction of multiple, separate sources of individual-differences variance, the utility lies in having a small set of measures that predicts academic outcomes and can be used by various stakeholders (guidance counselors, parents, and the students themselves) for determining the best elective courses for student success and satisfaction. Although these trait complex investigations were useful for this purpose, combining variables across trait families does not allow one to answer the critical research questions about the relative contributions of different individual traits or their intermediate-level amalgamations (e.g., personality traits only or interest traits only) to the prediction of academic achievement. Therefore, for the current concerns, we present the results without direct consideration of the trait complexes across cognitive and non-ability measures.

## 2. Method

### 2.1. Participants

Recruitment letters were sent to approximately 7000 9th-grade students/parents at 15 schools in the greater Atlanta, Georgia, USA metropolitan area. The schools represent a cross-section of urban, suburban, and exurban demographic groups. Consent/assent forms were returned by 1148 parents/students. A total of 914 students participated in this study by completing the ability tests and questionnaires, beginning in the 9th grade. Data from four students were excluded because of a failure to follow instructions in completion of the 9th-grade ability or questionnaire materials, leaving a total of 910 student participants. At the conclusion of high school (12th grade), complete grade transcripts were available for *N* = 738 students. These numbers reflect attrition from students transferring away from the participating schools or from student drop-outs. Complete transcripts through four years of high school were available for 81.2% of the overall sample. Additional measures were administered to students in the 10th–12th grade, along with teacher assessments and parent questionnaires. A discussion of these additional assessments is beyond the scope of the current investigation, and thus they are not reported here.

### 2.2. Apparatus

Student assessments were completed over the Internet with locally-developed software that served test items and questionnaire items individually on the screen to the students. The students accessed the items through their Internet browsers (e.g., Internet Explorer, Firefox, Safari). Parallel items were created that would automatically display in low resolution (1024 × 768 pixels) for older desktop and laptop computers, or in high resolution (greater than 1024 × 767) format for more recently manufactured computers. Questionnaires were administered such that they allowed the students to work at their own pace. For the ability and knowledge tests, total test times were imposed by a timer on the server that halted the tests when the time limits were reached. Students were informed about the time limits prior to each test. Transcripts were obtained from the schools via paper or via computer files, and were manually transcribed into a common format for later analyses.

### 2.3. Student Assessments

*Ninth-Grade Ability Tests*. In the first wave of data collection (early in the 9th grade), students completed a battery of ability tests (CogAT, Lohman and Hagen 2003). The CogAT is composed of 9 tests that are combined to provide composite measures of verbal, quantitative and figural abilities, which are listed in Table 1. From these composites, an overall estimate of general ability was also constructed for some analyses by combining the three ability composites.

*Questionnaires.* In the 9th-grade assessment, students also completed a set of questionnaires that focused on assessing traits from the cognitive (self-concept, self-estimates of ability), personality, motivation, interest domains, and several miscellaneous scales. A list of the main trait measures is provided in Table 1.

In the 10th, 11th, and 12th grades, the questionnaires (including personality, interests, self-concept, self-estimates of abilities, and other scales) were re-administered to the students. In general, each of these measures showed the expected simplex-like pattern of correlations; that is, in time-ordered measures, the largest correlations are found in those sampled closest to one another in time (e.g., 9th and 10th grade), and the smallest correlations are found for those measures most distant in time (e.g., 9th and 12th grade). The means for various non-ability measures were generally stable from one year to the next. For example, for the personality measures, 9th-grade to 12th-grade stabilities were generally high (Neuroticism *r* = 0.62, Extroversion, *r* = 0.68, Openness, *r* = 0.66, agreeableness, *r* = 0.63, and Conscientiousness, *r* = 0.58, respectively). Correlations with various criteria were also generally stable, but a discussion of these results is not presented here for the sake of brevity.

### 2.4. Twelfth-Grade Ability and Domain Knowledge Assessments

For the final assessment, a set of criterion ability and knowledge tests was administered. We administered two tests representative of verbal and quantitative abilities (vocabulary and math), along with six domain knowledge tests that represented a cross-section of topics represented in the general high school curriculum (US History, Biology, Western Civilization, US Literature, Chemistry, and US Government—see Table 1). Each of these was presented in a power test format (easier items first, followed by more difficult items), but under timed-test constraints (12 min each for vocabulary and math, and 20 min each for the domain knowledge tests). All tests were administered over the Internet, with a multiple-choice response format. For each test, the items ranged from very easy (could be answered by all or almost all students) to highly difficult (equivalent to college-level domain knowledge).

*Transcripts.* At the end of the 12th grade, student transcripts were obtained from the schools. For each student, each course completed, the term in which it was completed, and the grade received were coded. Separate codes were used for the following: (1) remedial courses, (2) honors courses, (3) Advanced Placement and Dual College Enrollment, (4) whether the course was an elective (i.e., whether the course was required, required but allowed for a course selection, or entirely an elective). Grades were recorded on an A = 4 format, regardless of whether the course was remedial, honors, AP, or other. (That is, the GPA was *not* weighted by course level.)

*Procedure*. After receiving permission from the appropriate authorities (e.g., school principals, headmasters, county-level IRB/research administrators, etc., as prescribed by the schools), recruitment letters were sent to all parents and ninth-grade students of the 15 participating schools in the fall of 2007. Once parent consent and student assent forms (including agreement to participate in this study and to release school transcript records) were completed, students were instructed how to access the test and questionnaire materials online. Reminders were sent via e-mail or postal mail to students who had started the materials, but who had not completed the materials, and a final deadline of January was established for completion. Students received remuneration of USD 32 for the 9th-grade assessments and USD 16 for the 12th-grade assessments. After the end of the spring academic term in the students’ 12th-grade year, requests were made from the schools for cumulative transcripts of all students who had participated in this study, starting in the 9th grade, including those who may have transferred schools or otherwise not completed a high-school diploma.

## 3. Analysis and Results

For the current purposes, to evaluate the independent and incremental validity of cognitive and non-ability traits for academic achievement, we focus on five domains of predictor constructs: cognitive abilities, personality traits, vocational interests, self-concept and self-estimates of abilities, and motivational traits. There are, of course, many different ways to examine the role of these constructs in predicting academic achievement. As noted earlier, from an applied perspective, our original goal was to provide stakeholders with a small number of trait complex indicators that would help guide recommendations for student enrollment in elective courses. While aggregation across trait families is useful for such purposes, such a strategy does not answer basic research questions about the independent and incremental validity of individual trait families or the individual traits themselves for predicting individual differences in academic achievement. Thus, the analyses and results reported here pertain to directly addressing the individual trait correlates, and their interactions, in predicting key criterion measures of academic achievement, in order to address the cognitive and non-ability contributions to the prediction of academic achievement.

The results are provided in four sections. In the first section, descriptive statistics are provided for the predictor and criterion measures. In the second section, raw correlations between each of the predictor measures and criterion measures are displayed. In the third section, a series of hierarchical correlations are provided, with cognitive abilities and non-ability predictors for each of the ability and academic achievement criterion indicators. In addition, correlations are reported for the predictor measures and a subset of criterion measures, where ninth-grade academic performance has been partialled from the equation. In the fourth and final section, we provide information about the influence of the predictors for whether or not the students participated in the elective AP program across the 9th–12th-grade years.

### 3.1. Descriptive Statistics of Predictor and Criterion Measures

#### 3.1.1. Ability, Knowledge Tests

In Table 2, basic descriptive statistics (number of items, means, standard deviations, and highest scores) are shown for the ability tests administered in the ninth grade. The notable items from these statistics are that even though Level G of the CogAT was administered (which is the version that the test developers deemed suitable for “High-Ability” ninth-grade students and “Average” tenth-grade students), a significant number of students achieved perfect scores on each of the tests. Although fewer than about 5% of the sample performed at this level on any of the tests, the resulting ceiling effects on the distribution of scores means that computed correlations between these test scores and the criterion variables will *underestimate* the magnitude of association between the underlying trait measures and the criteria. That is, the correlations will be smaller than would have been obtained if the ceiling effects had not been present.

#### 3.1.2. Criterion Measures

Table 2 also contains descriptive statistics for the ability and knowledge-criterion tests administered in the 12th grade. Because all of the 12th-grade ability and domain knowledge tests were designed with a higher ceiling (equivalent to knowledge attained by top students after a full year of college instruction in each domain), none of the students attained perfect scores on any of the 12th-grade assessments. Finally, additional criterion measures included GPA (both cumulative across 9th–12th grade and 12th grade only) and the number of elective Advanced Placement courses completed during high school.

In general, the students who participated in this study tended to have relatively high GPA scores—the mean GPA was equivalent to a B+ on an A/F scale, and 12 of the participants had perfect (4.0) GPA scores.

Table 3 provides descriptive statistics for the non-ability predictor measures, including personality traits, interests, self-concept and self-estimates of abilities, and motivational traits. In general, although these measures have been previously studied with older samples (e.g., college/university students and older adults), the means, standard deviations, and internal consistency reliability indices indicate that the measures were reasonably well suited for this sample of ninth-grade students. Although the mean scores of most of the measures do not have any ‘inherent’ meaning (given that they represent averages of Likert-type scales), the self-estimates-of-abilities measures were administered with percentile scales (i.e., between 1 and 99). The mean scores for these variables indicate that, in general, these students, on average, rated themselves near the top quartile of verbal, math, science, and intelligence variables (consistent with college/university students—e.g., see Ackerman and Wolman 2007).

Supplementary Materials include the full matrix of correlations among predictor variables and between predictor and criterion measures, both for a constant sample with complete data (*N* = 178) and for the overall sample (*N* = 178 to *N* = 738), depending on the pairwise number of students with data for pairs of variables.

#### 3.1.3. Raw Correlations Between Predictors and Criteria

Table 4 shows raw correlations of the cognitive ability and non-ability trait measures administered in the ninth grade, with criterion measures administered in the 12th grade (vocabulary, math ability, domain knowledge composite), along with both 12th GPA (only) and cumulative GPA (average across 9th–12th grades) scores, and finally the total number of elective Advanced Placement courses completed across the high-school years. The correlations reported in this table were calculated using ‘pairwise’ selection, meaning that when a participant had both scores, the correlation was computed. Because of attrition in this study at the 12th-grade ability and knowledge tests, the sample size for these criteria was *N* = 254. However, for the remaining criteria that were derived from school records, the sample size was *N* = 738.

Reviewing these correlations, it is clear that both cognitive ability and non-ability measures have significant positive correlations with the various criteria. The only exceptions to this pattern were the personality traits of Neuroticism (modest negative correlations), self-discipline and Extroversion (near zero correlations), three interest themes (social, enterprising, and conventional), and three of the motivational traits (competitiveness, worry, and emotionality), which consistently failed to show substantial correlations with any of the criteria. Among these, worry and emotionality tend to correlate substantially positively with neuroticism.

The largest magnitude correlations with the criterion measures were, as expected, found for the cognitive ability predictor measures. Overall, these measures tended to account for more than 25% of the variance in abilities measured in the 12th grade and with the number of AP courses completed during high school. Correlations with GPA, especially with 12th-grade-only GPA tended to be smaller (roughly 10% of the variance shared). Self-concept and self-estimates of abilities were consistently positively related to all the criterion measures, but were of lower magnitude in comparison to the ability measures. One thing to keep in mind about such measures is that, in contrast to the ability measures, each of the self-concept/self-estimates of abilities are self-report measures and are made up of just a handful of items (typically 1–7 items/scale). Thus, it may be considered reasonably impressive that such brief self-report measures administered in ninth grade are nonetheless significant predictors of cognitive ability and academic achievement across the 4 years of high school.

Personality trait measures that have been previously shown to be positive predictors of academic achievement and ability (e.g., see Poropat 2009) also showed similar positive correlations in this longitudinal study—specifically traits related to “engagement” (von Stumm and Ackerman 2013), including Openness to Experience, Typical Intellectual Engagement, Numerical Preferences, Need for Achievement. Modest, but statistically significant correlations with criteria were also found for the investigative interest theme, and motivational traits of Desire to Learn and Mastery.

#### 3.1.4. Common and Unique Criterion Variance Accounted for

The critical questions for this investigation are addressed in a series of multiple regressions, presented in Table 5. In these analyses, we report the aggregate predictive validity of each family of trait measures (i.e., cognitive abilities, personality, interests, self-concept/self-estimates of abilities, and motivational traits) and the incremental predictive validity in a hierarchical regression. The decision of which order to enter sets of variables in the prediction of the criteria is somewhat arbitrary, but given the question of whether non-ability measures add to the prediction of ability and academic achievement criteria, it was clear that cognitive ability predictors should be entered first into the equation. Following the cognitive ability measures, we decided that traits that *might* develop earliest should be included next (e.g., first personality traits, then vocational interests, followed by self-concept/self-estimates of abilities, and finally motivational traits). Other researchers might choose different orderings, but the final step, Total *R*^2^ at the bottom of the table will be unchanged, once all of the families of predictor variables are entered into the regression equation.

For Step 1 of the hierarchical regression, the cognitive ability measures, as predicted, accounted for variance in all the criterion measures, ranging from a relatively modest 16.1% of the variance in 12th-grade math ability and 15.4% of 12th-grade GPA to 39.5% of 12th-grade vocabulary and 49.7% 12th-grade domain knowledge. In addition, abilities accounted for 36.6% of the variance in the number of AP courses enrolled in across the high school years. For Step 2, although personality traits accounted for substantial variance in vocabulary, domain knowledge, and AP participation *in isolation*, the incremental variance accounted for after cognitive abilities was significant but modest (roughly 5–10% of additional variance accounted for), except for a non-significant contribution to predicting math ability). Once cognitive ability and personality traits were entered into the prediction equation, Interests largely did not contribute to the prediction of the criterion variables, with the exception of math ability (accounting for 11.6% of the variance in 12th-grade math ability). Similarly, once the other families of non-ability trait measures were included, self-concept/self-estimates of abilities only provided significant incremental predictive validity for 12th-grade vocabulary (additional 7.0% of variance accounted for). Finally, motivational traits only added significant variance accounted for in the vocabulary ability variable (a modest 3.9%).

Overall, even though the non-ability variables did not individually provide large amounts of incremental predictive validity over the cognitive ability measures for the criterion variables, the final variance accounted for (*R*^2^) for each indicates that in the aggregate, the overall prediction of the 12th-grade ability and academic achievement variables were much better when including the non-ability measures. The inclusion of the non-ability measures increased the variance accounted for (beyond cognitive abilities) by 20% for vocabulary, 12% for math ability, 13% for domain knowledge, 11% for 12th-rade GPA, 17% for cumulative GPA, and 9% for the number of AP courses completed. Accounting for cognitive ability, personality, interests, self-concept and motivational trait measures indicated that anywhere from 26.2% to 62.7% of the individual-differences variance was accounted for in ability and academic achievement indicators across 4 years of high-school experience.

Additional analyses were performed on the influence of cognitive and non-ability measures in predicting 12th-grade outcome and ability measures, with ninth-grade academic performance entered first into the equation (essentially this consists of correlations between the predictor and criterion measures, with ninth- grade GPA partialled from the equation), shown in Table 6. Included are correlations with vocabulary, math ability and domain-knowledge criteria. Unsurprisingly, the ability partial correlations are substantial, indicating that ninth-grade measured abilities account for criterion variance even when taking ninth-grade academic performance into account. The personality traits of Openness and TIE also accounted for significant variance in 12th-grade vocabulary and domain-knowledge criteria. Academic appetitive interests (realistic, investigative, and artistic interests) had small positive, but significant correlations with the ability and domain-knowledge criteria, as did self-concept and self-estimates-of-abilities variables. Finally, appetitive motivational traits of Desire to Learn and Mastery had positive correlations with domain-knowledge, and aversion-related motivational traits (worry and emotionality) had negative correlations with domain knowledge, even with ninth-grade academic performance statistically controlled.

A final analysis presented here pertains to the students’ decisions to participate in the AP program (coded as 0 = no, 1 = yes). That is, of the *N* = 738 students from whom we had complete transcripts, roughly 20% of them chose not to complete *any* AP courses. Typically, a decision to participate in the AP program reflects an interest in attending college/university study, because of the potential for obtaining college course credit from passing the follow-on AP tests and attaining passing scores, for which completing the AP course is usually a prerequisite. The analysis provided in Table 7 provides the correlations between the various trait measures and the number of AP courses completed, and also reports a *t*-test between the two groups that differed on whether the students completed *any* AP courses. The Cohen’s effect size difference between the groups provides another window into the role of cognitive ability and non-ability measures on the *choices* made by the students during their high school careers. Although again cognitive abilities evidenced the largest effect sizes (over *d* = 0.80), substantial effect sizes were also observed for a wide range of self-concept/self-estimates of abilities (as large as *d* = 0.64), and substantial effects for personality traits of Typical Intellectual Engagement (*d* = 0.55), Numerical Preferences *d* = 0.40), Need for Achievement (*d* = 0.38), Openness to Experience (*d* = 0.35), and the motivational traits of Desire to Learn (*d* = 0.33) and Other-Oriented Goals (*d* = 0.30).

## 4. Discussion and Conclusions

The results of this study indicate that, from a construct validity perspective, measures of cognitive ability continue to be well suited to the prediction of indicators of academic achievement in a longitudinal investigation across the secondary school years. Indicators of grades, abilities, and participation in advanced elective courses were all well predicted by a battery of cognitive/intellectual ability measures. Personality trait measures, although they were mostly not designed for the purpose of predicting academic achievement, also showed incremental predictive validity above the variance accounted for by cognitive ability measures. Measures of vocational interests and motivational traits provided much less robust incremental validity after cognitive abilities and personality traits were entered into the prediction equation. Measures of academic self-concept and self-ratings of abilities provide some incremental validity after abilities and personality traits were entered, which bolsters the notion that an individual’s self-concept does materially affect academic choices and achievement, over and above the other families of traits (e.g., see Marsh et al. 2018).

As noted earlier, previous empirical studies have not simultaneously sampled such a wide range of robust indicators of cognitive ability *and* non-ability constructs in a single longitudinal study of secondary school students, along with extensive measures of academic achievement indicators. Although other studies have individually pointed to the influences of particular abilities, personality, self-concept, and motivational traits on academic achievement indicators (sometimes with one or two families of traits), none have provided the basis to examine such a large number of constructs at the same time, which allowed for the evaluation of the common and unique variance contributions of these constructs in one sample (though see Lavrijsen et al. 2022, for a study of seventh graders with several of these types of measures).

In addition, claims that “personality”, “interests”, or “motivational traits” predict academic achievement are apparently over-broad. Several of the trait measures administered in this study were either non-significantly related to academic achievement indictors or had such small effect sizes as to be essentially unimportant for predicting traditional measures of individual differences in academic achievement.

Although these empirical results support the proposition that academic achievement is predicted partially by non-ability measures, it is important to reiterate that such measures are largely not theoretically linked to academic behaviors. With the exception of ‘engagement’-related variables, such as personality traits, which are thought to be most highly associated with *typical* behaviors, cognitive ability traits are most highly associated with *maximal* performance (e.g., see Ackerman 1994; Cronbach 1949; for a discussion, see Goff and Ackerman 1992). Given that there are few theories that postulate causal linkages between traits such as “agreeableness” or “extroversion” and academic achievement, at least at the level of secondary education, it should not be surprising that measures of such traits failed to account for individual differences in academic achievement. In contrast, re-conceptualizing some personality traits in a ‘maximal’ framework might yield a closer correspondence between personality and academic achievement. For example, traditional self-report measures of extroversion and introversion ask individuals about their preferences (e.g., “I enjoy giving presentations in class” and “I enjoy studying alone in a quiet room”), but they fail to ask about the individual’s flexibility or capability (e.g., “I am capable of giving a good presentation in class, even if I would prefer to listen to a lecture” or “I am capable of studying alone in a quiet room, even though I would rather go to a social event”) for typically extroverted or introverted behaviors (see discussion by Ackerman 1994). In this sense, flexibility of behaviors may be more important than an individual’s desired behaviors, especially in a context such as secondary school in which they have limited control of the environment. Future research along these lines may prove to be especially productive in linking cognitive ability and non-ability constructs in education.

As with any empirical study, there are limitations of the current investigation. Letters of invitation were sent to a large sample of students in schools both in urban and suburban environments, but the volunteer participants were a relatively modest sample of the larger population. In addition, because this study was originally concerned with students who had post-secondary educational aspirations, it is likely that the sample under-represented poor performing and struggling students. The fact that only 20% of the current sample did not complete any AP courses is consistent with the notion that this sample over-represented high-achieving students. It was not possible to obtain academic achievement records for students who did not participate in this study, so it was not possible to statistically adjust the correlations between predictors and criteria for restriction of range in talent (e.g., the cognitive ability measures). Given the higher correlations between the cognitive ability measures and academic achievement, compared to the non-ability measures, it might be a reasonable conjecture that the non-ability measures would have even less incremental validity for predicting academic achievement, if it were possible to either sample the student population more broadly or statistically adjust for restriction of range in talent. Finally, over the course of the four-year study, many students dropped out of this study, such that only a moderate-sized sample completed the 12th-grade assessments. This latter limitation, though, did not pertain to the GPA data, as those were provided for all of the sample (who had not dropped out or moved away), rather than just those students who completed the 12th-grade assessments.

From the results of this investigation, we conclude the following:

1. As expected, cognitive abilities assessed in ninth grade accounted for the largest portion of individual differences variance in 12th-grade abilities, grades, and the number of elective academic (AP) courses enrolled in during secondary school.
2. Non-ability trait measures, including personality traits, academically oriented vocational interests, and appetitive motivational traits assessed in ninth grade, also accounted for significant and meaningful variance in the 12th-grade criterion measures of academic achievement.
3. Once individual differences in cognitive abilities variance were accounted for (ranging from 15 to 50% of variance in criterion future measures of academic achievement), non-ability traits accounted for roughly 10–20% of incremental variance across the criterion variables. Although individual and, to some extent, joint investigations of non-ability trait predictors of academic achievement indictors have found similar levels of incremental validity over cognitive ability measures, the results of this study suggest that the 10–20% of incremental variance accounted for may represent a large degree of commonality among these non-ability measures that is only apparent when all families of traits are measured.
4. One of the most intriguing and unique aspects of the reported results is the influence of non-ability measures, especially those associated with ‘engagement’ and self-concept/self-ratings of abilities on whether or not students enrolled in *any* elective Advanced Placement courses, and if so, how many such courses were completed during the course of secondary education. These results are consistent with the theoretical proposition that non-ability traits are most likely to be valid predictors when the *environmental press* is low (or lower), in comparison to instructional environments where students are required to complete courses such as in basic science, math, and language courses that all students must complete, in order to receive a high-school diploma.
5. Even with a wide battery of cognitive and non-ability measures, there remains substantial individual differences variance in academic achievement criteria, suggesting that there are untapped sources of variance yet to be discovered.

## Figures and Tables

**Table 1:** Description of Measures Administered.

Ability and Domain Knowledge Assessments
**9th-Grade Ability Assessments**
The Cognitive Abilities Test (*CogAT*) battery, Level G, Form 6, was administered (Lohman and Hagen 2003). The test is composed of 9 tests, three tests for each of the Verbal, Quantitative, and Figural (Nonverbal) batteries.
*Verbal Battery*. In the *Verbal Classification* test, students are provided with items composed of three words that are related in some fashion, and they are to choose a fourth item that is also related in the same way from a set of options. In the *Sentence Completion* test, a sentence is presented with a blank instead of a word in the sentence. The student is instructed to select a word from a list of options that “best” completes the sentence. The *Verbal Analogies* test is a standard four-term analogy test, which takes the form of A ⟶ B: C ⟶ ?, with a set of options from which the student selects the one option that matches the relationship A is to B as C is to?
*Quantitative Battery*. In the *Quantitative Relations* test, each item starts with two given propositions or statements (e.g., I. 1/2 and II. 1/4) and then the student must choose among a set of answer choices that describe the relations among the propositions or statements (e.g., A. I is greater than II; B. II is greater than I; C. I and II are equal). The *Number Series* test provides the student with four numbers that have a consistent relationship among them (e.g., 1 3 5 7) and the student must select the next number that would fit within the sequence (in this example of increasing odd numbers, the next number would be 9). In the *Equation Building* test, the items are composed of a set of numbers and mathematical operators (e.g., +− ×÷). The student is instructed to combine the provided numbers and operators to “build” an equation in which the answer choice would follow an “=” sign.
*Nonverbal (Figural) Battery*. The first test of this battery is the *Figure Classification* test. In this test, students are provided with three figures that are alike in some way. They must choose a fourth item from a set of options that also has the same shared characteristic(s) of the first three figures. The *Figure Analogies* test is composed of standard four-term analogy problems, but the items are polygons made up of figures, instead of words. The *Figure Analysis* test is a paper-folding test. Each problem shows a plane view of a piece of paper that is folded in different ways, then a hole is punched in one place. The student has to find the option that indicates where the holes would appear when the page is completely unfolded.
**12th-Grade Ability and Domain Knowledge Assessments**
*Ability*
*Vocabulary*. ETS Extended Range Vocabulary Test. This is a classic vocabulary test. Individuals are presented with a word and must choose the word that most closely matches it (ETS kit: Ekstrom et al. 1976). *Math Ability*. This is a wide range test of mathematical abilities and knowledge, from simple computation to algebra, geometry, and other advanced topics. (Source: D. F. Lohman; see Ackerman and Kanfer 1993).
*Domain Knowledge Assessments* (source: Ackerman and Rolfhus 1999; Rolfhus and Ackerman 1999)
*Biology*. A broad range of biology, at the cellular, organismal, and ecological levels. Questions range from an understanding of food chains to the function of meiosis and mitosis. *Chemistry*. The content of a first year college course in chemistry, from the structure of the atom to standard laboratory procedures. *U.S. Government*. The structure of U.S. government, function of various government units, and the American political system. Typical questions ask about the significance of the Roe v. Wade decision of 1973 or the nature of the President’s veto power. *U.S. History*. U.S. history from pre-Revolutionary times to the present. Fifty percent of this test was derived from two College Board examinations, American History I: Early Colonization to 1877 and American History II: 1865 to the Present. *U.S. Literature*. A broad range of U.S. writers, playwrights and poets from Revolutionary times to the present. Questions require identification of authors and works from Walt Whitman to Kurt Vonnegut, and an understanding of literary styles and movements such as transcendentalism. *Western Civilization*. Major political, philosophical, and economic events in Europe from Ancient Greece to the Cold War. Typical items relate to the French Revolution or the genesis of World War II.
**II. Self-Report Measures**
Our assessments of non-ability constructs largely focused on existing broad constructs of personality, vocational interests, self-concept, and motivational traits, rather than on education-specific constructs, because our main interest was to relate robust constructs that apply generally to achievement settings across a wide range of applications.
*Personality*
*NEO-FFI*. The NEO-FFI (FFI stands for Five-Factor Inventory; Costa and McCrae 1992) was included to represent broad personality markers. This inventory is composed of 60 items to measure five factors: *Neuroticism, Extroversion, Openness to Experience, Conscientiousness,* and *Agreeableness*.
*Need for Achievement* and *Self-Discipline*. Two scales were administered from the International Personality Item Pool (IPIP; Goldberg 2008). The Need for Achievement scale was composed of 10 items; the Self-Discipline scale was composed of 9 items.
*Typical Intellectual Engagement (TIE)*. A shortened 12-item scale, based on the 59-item Goff and Ackerman (1992) scale was administered. Sample items are “There are few topics that bore me”, and “I read a great deal.” (Note: The TIE has been shown to have substantial overlap with a measure of need for Cognition; see, e.g., Woo et al. 2007).
*Numerical Preferences*. A shortened version adapted from the Viswanathan (1993) Preference for Numerical Information scale) was administered. This scale is composed of 11 items and measures an individual’s attraction or aversion to working with numerical or mathematical tasks.
*Vocational Interests*
The 90-item Unisex Edition of the American College Testing Interest Inventory (UNIACT; Lamb and Prediger 1981) was used to assess interest themes identified by Holland (1973) as: Realistic (“accounting”), Investigative (“studying Physics”), Artistic (“compose music”), Social (“run focus groups”), Enterprising (“entertain others”), and Conventional (“repair computers”).
*Self-Concept and Self-Estimates of Ability*
*Self-Concept*. Self-concepts for abilities and academic pursuits were assessed with a 21-item self-report questionnaire (Ackerman et al. 1995; Kanfer et al. 1996). The areas assessed were self-concepts of verbal, math, and science domains (e.g., “I can recognize correct English usage [i.e., grammar and punctuation]” “I understand concepts in my Sciences courses”).
*Self-Estimates of Ability*. For self-ratings of ability, a 14-item questionnaire was used (Ackerman et al. 1995). The items represent five scales of broadly described abilities, aptitudes, or skills, namely verbal, math, science, and general ability. Participants respond with a self-evaluation relative to other persons their age (in terms of percentile rank from 1 to 99). For example, in the verbal domain, items include “Writing Ability” and “Reading Ability.”
*Motivational Traits*
To reiterate our earlier discussion, the focus of this research was on the trait determinants of academic achievement. As such, even though there are numerous other research efforts on motivational factors in the educational context, we were mainly concerned with stable trait aspects of motivation, and not with situational or interventional variables. In conjunction with related personality traits, we assessed key appetitive and aversive aspects of motivational traits.
The short-form of the Motivational Trait Questionnaire (MTQ; Kanfer and Ackerman 2000; see also Heggestad and Kanfer 2000; Kanfer and Heggestad 1997) is a 48-item measure that contains six scales. The scales represent markers for three underlying motivational trait factors: (1) approach-oriented motivation (Desire to Learn, Mastery); (2) competitive excellence (other-referenced goals, competitiveness), and (3) aversion-related motivational traits (worry, emotionality).

**Table 2:** Descriptive statistics. Ninth-grade ability and 12th-grade ability/knowledge tests.

		Total # Items	Mean	Standard Deviation	Highest Score	(# of Perfect Scores)
*CogAT (9th-Grade Administration)*						
1.	Equation Building	15	9.33	3.73	15	24
2.	Figure Analogies	25	16.30	4.70	25	4
3.	Figure Analysis	15	9.52	3.79	15	46
4.	Figure Classification	25	17.20	5.36	25	16
5.	Number Series	20	14.38	4.20	20	41
6.	Quantitative Relations	25	18.55	4.26	25	22
7.	Sentence Completion	20	14.69	3.28	20	13
8.	Verbal Analogies	25	16.31	5.05	25	7
9.	Verbal Classification	20	13.09	3.99	20	9
*12th-Grade Ability and Domain Knowledge Tests*						
1.	Vocabulary	48	17.03	7.55	43.00	0
2.	Math Ability	32	9.21	3.22	15.75	0
3.	US History	123	56.53	15.79	99.50	0
4.	Biology	73	24.04	9.23	54.50	0
5.	Western Civilization	100	32.26	10.36	63.75	0
6.	US Literature	79	38.63	11.19	69.00	0
7.	Chemistry	58	18.98	7.33	41.75	0
8.	US Government	81	30.13	11.83	62.25	0
Grade Point Average						
1.	12th grade only	3.33	0.50	4.0	44	
2.	Cumulative 9th–12th	3.30	0.45	4.0	12	
AP Courses						
1.	Total Number Completed		7.33	6.19		[max = 27.]
2.	Participation (# ≥ 1) [No = 151, Yes = 587]					

AP = Advanced Placement; CogAT = Cognitive Abilities Test.

**Table 3:** Predictor variables—descriptive statistics and internal consistency reliability (alpha).

	#Items	Mean	sd	α
*Personality Traits*				
Neuroticism	12	3.30	0.86	0.83
Extroversion	12	4.34	0.67	0.78
Openness to Experience	12	3.71	0.63	0.64
Agreeableness	12	4.15	0.63	0.73
Conscientiousness	12	4.16	0.70	0.83
TIE	12	3.89	0.78	0.79
Need for Achievement	10	4.35	0.73	0.83
Self-Discipline	9	3.62	0.89	0.86
Numerical Preferences	11	4.07	0.98	0.90
*Interests*				
Realistic	15	3.13	0.92	0.89
Investigative	15	3.47	1.04	0.92
Artistic	15	3.86	1.04	0.90
Social	15	4.22	0.76	0.85
Enterprising	15	3.51	0.88	0.89
Conventional	15	2.80	0.97	0.92
*Self-Concept and Self-Estimates of Abilities*				
Verbal Self-Concept	7	4.58	0.92	0.86
Math Self-Concept	7	4.52	1.07	0.92
Science Self-Concept	7	4.36	1.03	0.92
Self-Estimate Verbal	6	74.95	16.38	0.85
Self-Estimate Math	5	72.49	19.68	0.91
Self-Estimate Science	1	68.12	26.53	-
Self-Estimate Intelligence	1	82.68	17.49	-
*Motivational Traits*				
Desire to Learn	8	4.32	0.76	0.82
Mastery	8	4.23	0.84	0.86
Other-Oriented Goals	7	4.01	0.91	0.83
Competitiveness	6	3.69	1.05	0.86
Worry	10	3.59	0.92	0.85
Emotionality	9	3.21	0.87	0.80

TIE = Typical Intellectual Engagement.

**Table 4:** Predictor correlates with senior-year vocabulary, math ability, domain knowledge ^1^, GPA (12th grade and cumulative) and total number of Advanced Placement courses completed.

	Vocabulary	Math	Domain	12Grade	Cumu.	Adv. Placement
9th-Grade Measures		Ability	Knowledge	GPA	GPA	Total
*Abilities*						
Verbal	0.644 **	0.317 **	0.618 **	0.375 **	0.484 **	0.531 **
Quantitative	0.349 **	0.383 **	0.496 **	0.317 **	0.437 **	0.548 **
Figural	0.368 **	0.431 **	0.419 **	0.327 **	0.440 **	0.471 **
*General Composite* ^2^	0.525 **	0.447 **	0.579 **	0.387 **	0.516 **	0.578 **
*Personality Traits*						
Neuroticism	−0.089	−0.059	−0.173 **	−0.060	−0.070	−0.116 **
Extroversion	−0.103	0.017	−0.087	−0.002	−0.072	−0.074 *
Openness to Experience	0.339 **	0.090	0.269 **	0.025	0.088 *	0.236 **
Agreeableness	0.042	0.047	0.074	0.160 **	0.172 **	0.039
Conscientiousness	0.058	0.076	0.113	0.184 **	0.221 **	0.087 *
TIE	0.330 **	0.175 **	0.314 **	0.073 *	0.180 **	0.304 **
Need for Achievement	0.202 **	0.165 *	0.239 **	0.185 **	0.290 **	0.230 **
Self-Discipline	−0.059	0.008	0.030	0.046	0.078	−0.026
Numerical Preferences	0.129 *	0.264 **	0.204 **	0.147 **	0.236 **	0.253 **
*Interests*						
Realistic	0.062	0.249 **	0.094	−0.005	−0.022	0.096 **
Investigative	0.220 **	0.229 **	0.205 **	0.066	0.112 **	0.191 **
Artistic	0.230 **	0.048	0.176 **	0.045	0.044	0.127 **
Social	0.001	0.031	0.009	0.021	−0.008	0.017
Enterprising	0.015	−0.076	0.014	−0.075 *	−0.083 *	0.067
Conventional	0.023	0.083	0.018	−0.019	−0.001	0.065
*Self-Concept and Self-Estimates of Abilities*						
Verbal Self-Concept	0.285 **	0.004	0.212 **	0.038	0.081 *	0.152 **
Math Self-Concept	0.097	0.294 **	0.230 **	0.233 **	0.316 **	0.290 **
Science Self-Concept	0.233 **	0.228 **	0.328 **	0.142 **	0.222 **	0.288 **
Self-Estimate Verbal	0.380 **	0.034	0.356 **	0.059	0.129 **	0.246 **
Self-Estimate Math	0.181 **	0.291 **	0.317 **	0.216 **	0.322 **	0.335 **
Self-Estimate Science	0.254 **	0.285 **	0.359 **	0.150 **	0.250 **	0.137 **
Self-Estimate Intelligence	0.215 **	0.121	0.345 **	0.100 **	0.189 **	0.318 **
*Motivational Traits*						
Desire to Learn	0.215 **	0.164 *	0.304 **	0.056	0.138 **	0.226 **
Mastery	0.091	0.177 **	0.158 **	0.103 **	0.202 **	0.145 **
Other-Oriented Goals	0.072	0.210 **	0.120 *	0.102 **	0.168 **	0.162 **
Competitiveness	0.081	0.194 **	0.079	0.007	0.012	0.064
Worry	0.017	0.023	−0.055	0.072	0.048	0.007
Emotionality	−0.088	−0.001	−0.130 *	−0.030	−0.060	−0.094 *

Note: * *p* < .05; ** *p* < .01; TIE = Typical Intellectual Engagement; AP = Advanced Placement. ^1^ Domain knowledge is a composite of the following tests: US History, US Government, Literature, Biology, Chemistry, and Western Civilization. ^2^ General composite is a unit-weighted z-score composite of Verbal, Math, and Figural tests; italics are added to emphasize that this composite is not statistically independent of the other ability tests.

**Table 5:** Hierarchical regression for vocabulary, math, domain knowledge, 12th-grade GPA, cumulative GPA, #AP courses.

		Vocabulary	Math	Domain	12Grade	Cumu.	Adv. Placement
9th-Grade Measures			Ability	Knowledge	GPA	GPA	Total
Step in Hierarchical Regression							
1.	Ability						
	*R* ^2^	0.395 **	0.161 **	0.497 **	0.154 **	0.274 **	0.366 **
	Total *R*^2^	0.395 **	0.161 **	0.497 **	0.154 **	0.274 **	0.366 **
2.	Personality						
	*R*^2^ in isolation	0.278 **	0.101 *	0.291 **	0.096 **	0.096 **	0.242 **
	*R*^2^ to Add	0.062 *	0.019 ns	0.061 *	0.058 **	0.105 **	0.069 **
	Total *R*^2^	0.457 **	0.180 **	0.558 **	0.213 **	0.379 **	0.435 **
3.	Interests						
	*R*^2^ in isolation	0.124 **	0.126 **	0.067 *	0.056 *	0.070 *	0.060 **
	*R*^2^ to Add	0.031 ns	0.116 **	0.010 ns	0.019 ns	0.022 **	0.002 ns
	Total *R*^2^	0.488 **	0.296 **	0.568 **	0.232 **	0.401 **	0.437 **
4.	Self-Concept and Self-Estimates of Abilities						
	*R*^2^ in isolation	0.199 **	0.170 **	0.280 **	0.067 **	0.068 **	0.199 **
	*R*^2^ to Add	0.070 **	0.051 ns	0.043 *	0.021 ns	0.024 *	0.017 ns
	Total *R*^2^	0.558 **	0.346 **	0.611 **	0.253 **	0.425 **	0.454 **
5.	Motivational Traits						
	*R*^2^ in isolation	0.059 ns	0.060 ns	0.148 **	0.024 ns	0.024 ns	0.073 **
	*R*^2^ to Add	0.039 *	0.040 ns	0.015 ns	0.009 ns	0.015 *	0.005 ns
	Total *R*^2^	0.597 **	0.387 **	0.627 **	0.262 **	0.440 **	0.460 **

* *p* < .05; ** *p* < .01; ns = not significant.

**Table 6:** Predictor correlates with senior-year vocabulary, math ability, domain knowledge and 12th-grade GPA, with ninth-grade GPA partialled out.

	Vocabulary	Math	Domain	12Grade
9th-Grade Measures		Ability	Knowledge	GPA
*Abilities*				
Verbal	0.535 **	0.137	0.547 **	0.189 *
Quantitative	0.152 *	0.195 **	0.386 **	0.027
Figural	0.162 *	0.267 **	0.245 **	0.024
*General Composite* ^2^	0.342 **	0.258 **	0.460 **	*0.095*
*Personality Traits*				
Neuroticism	0.130	−0.065	−0.262 **	−0.070
Extroversion	−0.116	0.001	−0.033	0.066
Openness to Experience	0.273 **	0.046	0.224 **	0.025
Agreeableness	−0.003	0.000	0.003	0.101
Conscientiousness	0.007	−0.045	0.029	0.110
TIE	0.249 **	0.064	0.248 **	0.011
Need for Achievement	0.110	0.032	0.160 *	0.046
Self-Discipline	−0.082	−0.071	−0.005	0.011
Numerical Preferences	0.062	0.159 *	0.129	0.008
*Interests*				
Realistic	0.009	0.249 **	0.155 *	−0.032
Investigative	0.161 *	0.192 **	0.210 **	0.047
Artistic	0.167 *	−0.015	0.134	0.063
Social	−0.040	0.012	0.020	0.028
Enterprising	0.088	−0.094	0.140	0.030
Conventional	0.061	0.080	0.095	−0.030
*Self-Concept and Self-Estimates of Abilities*				
Verbal Self-Concept	0.288 **	−0.071	0.183 *	−0.002
Math Self-Concept	−0.012	0.224 **	0.140	0.098
Science Self-Concept	0.157 *	0.202 **	0.351 **	0.076
Self-Estimate Verbal	0.309 **	−0.113	0.255 **	0.006
Self-Estimate Math	0.030	0.175 *	0.177 *	0.088
Self-Estimate Science	0.147	0.216 **	0.300 **	0.071
Self-Estimate Intelligence	0.071	−0.100	0.227 **	0.060
*Motivational Traits*				
Desire to Learn	0.143	0.084	0.280 **	0.009
Mastery	0.000	0.103	0.154 *	−0.023
Other-Oriented Goals	−0.020	0.111	0.034	−0.050
Competitiveness	0.031	0.083	0.138	0.013
Worry	−0.027	−0.045	−0.159 *	−0.081
Emotionality	−0.073	−0.016	−0.192 *	−0.046

* *p* < .05; ** *p* < .01. ^2^ General composite is a unit-weighted z-score composite of Verbal, Math, and Figural tests; italics are added to emphasize that this composite is not statistically independent of the other ability tests.

**Table 7:** Correlates of traits measured in 9th-grade and AP participation across high school years (listwise deletion).

	#AP Courses	Participation	
		*t*	Cohen’s D
**Assessed during 9th grade**			
*Abilities*			
Verbal	0.517 **	9.14 **	1.07
Quantitative	0.538 **	8.89 **	1.04
Figural	0.446 **	7.14 **	0.84
*General Composite* ^2^	0.559 **	*9.44*	*1.11*
*Personality Traits*			
Neuroticism	−0.149 **	−1.56 ns	−0.14
Extroversion	−0.107 *	−0.78 ns	−0.07
Openness to Experience	0.240 **	3.88 **	0.35
Agreeableness	0.020	0.30 ns	0.03
Conscientiousness	0.090 *	1.22 ns	0.11
TIE	0.320 **	6.05 **	0.55
Numerical Preferences	0.269 **	4.34 **	0.40
Need for Achievement	0.236 **	4.12 **	0.38
Self-Discipline	−0.011	−0.79 ns	−0.07
*Interests*			
Realistic	0.055	−0.56 ns	−0.05
Investigative	0.188 **	2.64 **	0.24
Artistic	0.098 *	1.97 *	0.18
Social	−0.021	−0.79 ns	−0.07
Enterprising	0.037	−0.82 ns	−0.08
Conventional	0.093 *	−0.51 ns	−0.05
*Self-Concept and Self-Estimates of Abilities*			
Verbal Self-Concept	0.193 **	3.32 **	0.30
Math Self-Concept	0.294 **	6.04 **	0.55
Science Self-Concept	0.297 **	6.49 **	0.59
Self-Estimate Verbal	0.273 **	5.15 **	0.47
Self-Estimate Math	0.343 **	6.75 **	0.61
Self-Estimate Science	0.312 **	6.68 **	0.61
Self-Estimate Intelligence	0.314 **	7.07 **	0.64
*Motivational Traits*			
Desire to Learn	0.261 **	3.65 **	0.33
Mastery	0.162 **	2.34 *	0.21
Other-Oriented Goals	0.115 **	3.24 **	0.30
Competitiveness	0.044	1.00 ns	0.09
Worry	−0.042	1.29 ns	0.12
Emotionality	−0.127 **	−1.38 ns	−0.13

TIE = Typical Intellectual Engagement, * *p* < .05; ** *p* < .01, ns = not significant. ^2^ General composite is a unit-weighted z-score composite of Verbal, Math, and Figural tests; italics are added to emphasize that this composite is not statistically independent of the other ability tests.

## Data Availability

The data presented in this study are available on request from the corresponding author. Individual-level data are unavailable due to privacy restrictions.

## Supplementary Materials

The following supporting information can be downloaded at: https://www.mdpi.com/article/10.3390/jintelligence13070079/s1, Table S1: Supplementary table of intercorrlations among predictor and criterion variables. *N* = 178 participants with complete records of predictor and criterion variables. Table S2: Supplementary table of intercorrlations among predictor and criterion variables. Number of participants with data are indicated for each pairwise correlation in parentheses. Table S3: Abbreviations in Table 1 and Table 2.
