# Supplementary material for: Cognitive Ability and Non-Ability Trait Predictors of Academic Achievement: A Four-Year Longitudinal Study
Source: J Intell. 2025 Jun 30;13(7):79. doi: 10.3390/jintelligence13070079 (PMC12295058; doi:10.3390/jintelligence13070079)
Supplement: Supplementary file 1 [file jintelligence-13-00079-s001.zip › jintelligence-3513870-supplementary.pdf]

Table S1. Supplementary table of intercorrlations among predictor and criterion variables. N = 178 participants with complete records of predictor and criterion variables.

- - Correlation Coefficients - -

	VERBAL	QUANT	FIGURAL	GENERAL	N	E	O	A	C	TIE
VERBAL	1.0000	.4906**	.5224**	.7846**	-.1536*	-.0599	.2577**	.1137	.1589*	.2380**
QUANT	.4906**	1.0000	.7141**	.8305**	-.1952**	.0792	.0940	.1345	.1128	.1465
FIGURAL	.5224**	.7141**	1.0000	.9140**	-.1587*	.0442	.1117	.1660*	.1657*	.2211**
GENERAL	.7846**	.8305**	.9140**	1.0000	-.1941**	.0220	.1814*	.1663*	.1773*	.2453**
N	-.1536*	-.1952**	-.1587*	-.1941**	1.0000	-.3763**	.0294	-.3163**	-.3471**	-.1916*
E	-.0599	.0792	.0442	.0220	-.3763**	1.0000	-.0139	.2335**	.2228**	.1621*
O	.2577**	.0940	.1117	.1814*	.0294	-.0139	1.0000	-.0802	-.0048	.5202**
A	.1137	.1345	.1660*	.1663*	-.3163**	.2335**	-.0802	1.0000	.2768**	.1177
C	.1589*	.1128	.1657*	.1773*	-.3471**	.2228**	-.0048	.2768**	1.0000	.3082**
TIE	.2380**	.1465	.2211**	.2453**	-.1916*	.1621*	.5202**	.1177	.3082**	1.0000
NACH	.2649**	.1977**	.2287**	.2735**	-.3326**	.3622**	.1266	.1924*	.7860**	.3992**
SELFD	-.0275	.0612	.0612	.0382	-.4074**	.2221**	-.0538	.2211**	.7821**	.3181**
NUMPR	.1373	.4238**	.3866**	.3691**	-.3191**	.2156**	.0512	.1999**	.2677**	.2409**
REALISTIC	.1090	.2149**	.2642**	.2376**	-.2960**	.1134	.0647	.1422	.1332	.2202**
INVEST	.1838*	.1734*	.2174**	.2302**	-.1337	-.1216	.3938**	.1424	.1807*	.4180**
ARTISTIC	.3214**	.0601	.1225	.2019**	.0512	.1654*	.4794**	.0177	.0930	.2267**
SOCIAL	.0992	.1268	.1533*	.1521*	-.2209**	.5522**	.1950**	.2492**	.2349**	.2987**
ENTERPRISE	.0711	.2131**	.2116**	.1953**	-.2840**	.4434**	.1685*	.0859	.3235**	.2932**
CONVENTION	.0187	.3348**	.3375**	.2744**	-.1623*	.1435	.0045	.0511	.1982**	.1905*
VERBALSC	.3089**	.1346	.1196	.2159**	-.0964	.2715**	.3956**	-.0270	.3358**	.4590**
MATHSC	.1481*	.4080**	.4509**	.4025**	-.3449**	.1946**	-.1109	.2106**	.3492**	.1593*
SCIENCSC	.2793**	.2057**	.2651**	.3000**	-.2299**	-.0145	.2202**	.0589	.3829**	.5227**
VERBALSE	.2903**	.1789*	.1651*	.2447**	-.2078**	.2284**	.2971**	-.0353	.3390**	.3963**
MATHSE	.1628*	.4706**	.4605**	.4301**	-.3773**	.1641*	-.0655	.2244**	.3085**	.1259
SCIENCESE	.2717**	.2117**	.2520**	.2919**	-.2729**	-.0631	.0797	.0769	.3337**	.3390**
GSE	.3105**	.2706**	.2434**	.3182**	-.2115**	.0761	.1697*	.0639	.3176**	.2212**
DTL	.2048**	.1484*	.2040**	.2244**	-.2628**	.1262	.4281**	.1416	.5660**	.5964**
MASTERY	.2245**	.2296**	.2428**	.2742**	-.3158**	.3931**	.1819*	.2104**	.6290**	.3507**
OTHER	.0374	.1113	.0681	.0800	.0410	.2031**	-.0487	-.1445	.1027	.0432
COMPET	.0267	.1951**	.0792	.1045	-.2594**	.4176**	.0224	-.1718*	.1506*	.1494*
WORRY	-.0566	-.0748	-.0756	-.0812	.6958**	-.2719**	-.0303	-.0940	-.2285**	-.1639*

EMOTION	-.0753	-.1152	-.1228	-.1239	.7394**	-.2384**	-.0583	-.2173**	-.3193**	-.2645**
VOCABULARY	.6278**	.3065**	.3131**	.4844**	-.1522*	-.0923	.3032**	.0860	.1374	.3161**
MATHABIL	.2918**	.3245**	.3838**	.3990**	-.0914	.0134	.0931	.0799	.0762	.1433
KNOWLEDGE	.6627**	.5248**	.4159**	.6107**	-.2653**	-.0112	.2615**	.1155	.1896*	.3263**
12THGPA	.4146**	.2699**	.2653**	.3688**	-.1046	.0727	.0994	.2038**	.2661**	.1408
CUMGPA	.5489**	.4068**	.3911**	.5226**	-.0845	.0350	.1392	.2208**	.3304**	.2190**
APTOTAL	.5175**	.5035**	.4203**	.5522**	-.1923*	-.0219	.2744**	.0810	.1573*	.3809**

	NACH	SELFD	NUMPR	REALISTIC	INVEST	ARTISTIC	SOCIAL	ENTERPRISE	CONVENTION	VERBALSC
VERBAL	.2649**	-.0275	.1373	.1090	.1838*	.3214**	.0992	.0711	.0187	.3089**
QUANT	.1977**	.0612	.4238**	.2149**	.1734*	.0601	.1268	.2131**	.3348**	.1346
FIGURAL	.2287**	.0612	.3866**	.2642**	.2174**	.1225	.1533*	.2116**	.3375**	.1196
GENERAL	.2735**	.0382	.3691**	.2376**	.2302**	.2019**	.1521*	.1953**	.2744**	.2159**
N	-.3326**	-.4074**	-.3191**	-.2960**	-.1337	.0512	-.2209**	-.2840**	-.1623*	-.0964
E	.3622**	.2221**	.2156**	.1134	-.1216	.1654*	.5522**	.4434**	.1435	.2715**
O	.1266	-.0538	.0512	.0647	.3938**	.4794**	.1950**	.1685*	.0045	.3956**
A	.1924*	.2211**	.1999**	.1422	.1424	.0177	.2492**	.0859	.0511	-.0270
C	.7860**	.7821**	.2677**	.1332	.1807*	.0930	.2349**	.3235**	.1982**	.3358**
TIE	.3992**	.3181**	.2409**	.2202**	.4180**	.2267**	.2987**	.2932**	.1905*	.4590**
NACH	1.0000	.6827**	.3124**	.1707*	.2453**	.2122**	.3600**	.4120**	.1967**	.4311**
SELFD	.6827**	1.0000	.2508**	.1910*	.1772*	-.0013	.1696*	.2775**	.1897*	.2211**
NUMPR	.3124**	.2508**	1.0000	.3872**	.2037**	-.0909	.1780*	.2148**	.5550**	-.0691
REALISTIC	.1707*	.1910*	.3872**	1.0000	.5097**	.2110**	.3839**	.3908**	.5149**	-.0982
INVEST	.2453**	.1772*	.2037**	.5097**	1.0000	.1908*	.2753**	.1478*	.2117**	.1118
ARTISTIC	.2122**	-.0013	-.0909	.2110**	.1908*	1.0000	.5116**	.3610**	-.0103	.5150**
SOCIAL	.3600**	.1696*	.1780*	.3839**	.2753**	.5116**	1.0000	.6331**	.2774**	.3589**
ENTERPRISE	.4120**	.2775**	.2148**	.3908**	.1478*	.3610**	.6331**	1.0000	.5899**	.3458**
CONVENTION	.1967**	.1897*	.5550**	.5149**	.2117**	-.0103	.2774**	.5899**	1.0000	.0199
VERBALSC	.4311**	.2211**	-.0691	-.0982	.1118	.5150**	.3589**	.3458**	.0199	1.0000
MATHSC	.3674**	.2825**	.7079**	.3795**	.1635*	-.1114	.1265	.2137**	.4792**	-.0317
SCIENCSC	.3913**	.3038**	.2592**	.2921**	.6045**	.0209	.1142	.1494*	.1816*	.2342**
VERBALSE	.4575**	.2903**	-.0316	.0322	.1573*	.4038**	.2824**	.3590**	.0800	.6841**
MATHSE	.3376**	.2936**	.5927**	.3734**	.1709*	-.0484	.1449	.2135**	.4513**	-.0699
SCIENCESE	.3662**	.3347**	.2196**	.3212**	.4985**	.0552	.0395	.0400	.1262	.1219
GSE	.3854**	.2599**	.0920	.2139**	.1889*	.3056**	.2073**	.2677**	.1973**	.3472**
DTL	.6613**	.4520**	.3006**	.2585**	.5453**	.1737*	.2781**	.2500**	.1856*	.3504**
MASTERY	.7891**	.4871**	.3197**	.1656*	.2824**	.1857*	.3703**	.3682**	.2202**	.3574**
OTHER	.2708**	.0625	.1444	.0081	.1254	.0159	.1098	.1407	.0685	.1392
COMPET	.3051**	.1585*	.1897*	-.0096	-.0014	-.0306	.1943**	.2088**	.0310	.1953**
WORRY	-.1639*	-.2615**	-.0989	-.1958**	-.0259	-.0276	-.1336	-.1922*	-.0106	-.0829
EMOTION	-.2760**	-.4111**	-.2197**	-.2256**	-.1179	.0307	-.1169	-.1836*	-.0620	-.0555
VOCABULARY	.2404**	-.0028	.1431	.0138	.1894*	.2132**	-.0082	.0601	.0623	.3347**
MATHABIL	.1580*	-.0016	.2233**	.2364**	.2163**	.0414	.0373	-.1050	.0809	.0029
KNOWLEDGE	.3129**	.0859	.2189**	.1385	.2331**	.1915*	.0538	.0941	.0892	.2500**
12THGPA	.2374**	.1075	.1279	-.0194	.0985	.1368	.0632	-.0022	-.0157	.1032
CUMGPA	.3628**	.1616*	.1524*	-.0092	.1065	.1628*	.0599	-.0261	-.0162	.1918*

APTOTAL	.2900**	.0412	.2601**	.1297	.2061**	.1693*	.1424	.1711*	.1708*	.2420**

	MATHSC	SCIENCSC	VERBALSE	MATHSE	SCIENCESE	GSE	DTL	MASTERY	OTHER	COMPET
VERBAL	.1481*	.2793**	.2903**	.1628*	.2717**	.3105**	.2048**	.2245**	.0374	.0267
QUANT	.4080**	.2057**	.1789*	.4706**	.2117**	.2706**	.1484*	.2296**	.1113	.1951**
FIGURAL	.4509**	.2651**	.1651*	.4605**	.2520**	.2434**	.2040**	.2428**	.0681	.0792
GENERAL	.4025**	.3000**	.2447**	.4301**	.2919**	.3182**	.2244**	.2742**	.0800	.1045
N	-.3449**	-.2299**	-.2078**	-.3773**	-.2729**	-.2115**	-.2628**	-.3158**	.0410	-.2594**
E	.1946**	-.0145	.2284**	.1641*	-.0631	.0761	.1262	.3931**	.2031**	.4176**
O	-.1109	.2202**	.2971**	-.0655	.0797	.1697*	.4281**	.1819*	-.0487	.0224
A	.2106**	.0589	-.0353	.2244**	.0769	.0639	.1416	.2104**	-.1445	-.1718*
C	.3492**	.3829**	.3390**	.3085**	.3337**	.3176**	.5660**	.6290**	.1027	.1506*
TIE	.1593*	.5227**	.3963**	.1259	.3390**	.2212**	.5964**	.3507**	.0432	.1494*
NACH	.3674**	.3913**	.4575**	.3376**	.3662**	.3854**	.6613**	.7891**	.2708**	.3051**
SELFD	.2825**	.3038**	.2903**	.2936**	.3347**	.2599**	.4520**	.4871**	.0625	.1585*
NUMPR	.7079**	.2592**	-.0316	.5927**	.2196**	.0920	.3006**	.3197**	.1444	.1897*
REALISTIC	.3795**	.2921**	.0322	.3734**	.3212**	.2139**	.2585**	.1656*	.0081	-.0096
INVEST	.1635*	.6045**	.1573*	.1709*	.4985**	.1889*	.5453**	.2824**	.1254	-.0014
ARTISTIC	-.1114	.0209	.4038**	-.0484	.0552	.3056**	.1737*	.1857*	.0159	-.0306
SOCIAL	.1265	.1142	.2824**	.1449	.0395	.2073**	.2781**	.3703**	.1098	.1943**
ENTERPRISE	.2137**	.1494*	.3590**	.2135**	.0400	.2677**	.2500**	.3682**	.1407	.2088**
CONVENTION	.4792**	.1816*	.0800	.4513**	.1262	.1973**	.1856*	.2202**	.0685	.0310
VERBALSC	-.0317	.2342**	.6841**	-.0699	.1219	.3472**	.3504**	.3574**	.1392	.1953**
MATHSC	1.0000	.3253**	.0193	.7907**	.2727**	.1892*	.2913**	.2789**	.1402	.1482*
SCIENCSC	.3253**	1.0000	.2303**	.2434**	.7274**	.2331**	.5765**	.3298**	.1214	.0347
VERBALSE	.0193	.2303**	1.0000	.2560**	.3419**	.6848**	.4042**	.3949**	.0776	.1320
MATHSE	.7907**	.2434**	.2560**	1.0000	.3900**	.4427**	.3114**	.3001**	.1016	.1495*
SCIENCESE	.2727**	.7274**	.3419**	.3900**	1.0000	.3961**	.4236**	.2652**	.0323	-.0085
GSE	.1892*	.2331**	.6848**	.4427**	.3961**	1.0000	.3772**	.2771**	.1088	.0733
DTL	.2913**	.5765**	.4042**	.3114**	.4236**	.3772**	1.0000	.7021**	.2246**	.2088**
MASTERY	.2789**	.3298**	.3949**	.3001**	.2652**	.2771**	.7021**	1.0000	.3032**	.3236**
OTHER	.1402	.1214	.0776	.1016	.0323	.1088	.2246**	.3032**	1.0000	.6516**
COMPET	.1482*	.0347	.1320	.1495*	-.0085	.0733	.2088**	.3236**	.6516**	1.0000
WORRY	-.2037**	-.1274	-.1093	-.1806*	-.1637*	-.1062	-.1409	-.1224	.2602**	-.1288
EMOTION	-.2435**	-.2283**	-.1982**	-.3084**	-.2882**	-.1991**	-.2847**	-.1804*	.1528*	-.2063**
VOCABULARY	.1060	.2333**	.3789**	.1684*	.2499**	.1985**	.2297**	.1358	.0451	.0530
MATHABIL	.3031**	.2667**	-.0071	.2801**	.3003**	.0336	.1678*	.2109**	.1588*	.0987
KNOWLEDGE	.2623**	.4093**	.3449**	.3219**	.3984**	.3555**	.3612**	.2947**	.1093	.1479*
12THGPA	.2374**	.1904*	.1523*	.2640**	.2238**	.2357**	.1496*	.1677*	.0470	.0444
CUMGPA	.2530**	.2327**	.2357**	.3216**	.2714**	.3180**	.2290**	.3174**	.1347	.0675

APTOTAL	.2900**	.3192**	.2790**	.3090**	.3178**	.3206**	.3105**	.2619**	.0699	.1103

	WORRY	EMOTION	VOCABULARY	MATHABIL	KNOWLEDGE	12THGPA	CUMGPA	APTOT
VERBAL	-.0566	-.0753	.6278**	.2918**	.6627**	.4146**	.5489**	.5175**
QUANT	-.0748	-.1152	.3065**	.3245**	.5248**	.2699**	.4068**	.5035**
FIGURAL	-.0756	-.1228	.3131**	.3838**	.4159**	.2653**	.3911**	.4203**
GENERAL	-.0812	-.1239	.4844**	.3990**	.6107**	.3688**	.5226**	.5522**
N	.6958**	.7394**	-.1522*	-.0914	-.2653**	-.1046	-.0845	-.1923*
E	-.2719**	-.2384**	-.0923	.0134	-.0112	.0727	.0350	-.0219
O	-.0303	-.0583	.3032**	.0931	.2615**	.0994	.1392	.2744**
A	-.0940	-.2173**	.0860	.0799	.1155	.2038**	.2208**	.0810
C	-.2285**	-.3193**	.1374	.0762	.1896*	.2661**	.3304**	.1573*
TIE	-.1639*	-.2645**	.3161**	.1433	.3263**	.1408	.2190**	.3809**
NACH	-.1639*	-.2760**	.2404**	.1580*	.3129**	.2374**	.3628**	.2900**
SELFD	-.2615**	-.4111**	-.0028	-.0016	.0859	.1075	.1616*	.0412
NUMPR	-.0989	-.2197**	.1431	.2233**	.2189**	.1279	.1524*	.2601**
REALISTIC	-.1958**	-.2256**	.0138	.2364**	.1385	-.0194	-.0092	.1297
INVEST	-.0259	-.1179	.1894*	.2163**	.2331**	.0985	.1065	.2061**
ARTISTIC	-.0276	.0307	.2132**	.0414	.1915*	.1368	.1628*	.1693*
SOCIAL	-.1336	-.1169	-.0082	.0373	.0538	.0632	.0599	.1424
ENTERPRISE	-.1922*	-.1836*	.0601	-.1050	.0941	-.0022	-.0261	.1711*
CONVENTION	-.0106	-.0620	.0623	.0809	.0892	-.0157	-.0162	.1708*
VERBALSC	-.0829	-.0555	.3347**	.0029	.2500**	.1032	.1918*	.2420**
MATHSC	-.2037**	-.2435**	.1060	.3031**	.2623**	.2374**	.2530**	.2900**
SCIENCSC	-.1274	-.2283**	.2333**	.2667**	.4093**	.1904*	.2327**	.3192**
VERBALSE	-.1093	-.1982**	.3789**	-.0071	.3449**	.1523*	.2357**	.2790**
MATHSE	-.1806*	-.3084**	.1684*	.2801**	.3219**	.2640**	.3216**	.3090**
SCIENCESE	-.1637*	-.2882**	.2499**	.3003**	.3984**	.2238**	.2714**	.3178**
GSE	-.1062	-.1991**	.1985**	.0336	.3555**	.2357**	.3180**	.3206**
DTL	-.1409	-.2847**	.2297**	.1678*	.3612**	.1496*	.2290**	.3105**
MASTERY	-.1224	-.1804*	.1358	.2109**	.2947**	.1677*	.3174**	.2619**
OTHER	.2602**	.1528*	.0451	.1588*	.1093	.0470	.1347	.0699
COMPET	-.1288	-.2063**	.0530	.0987	.1479*	.0444	.0675	.1103
WORRY	1.0000	.7425**	-.0153	-.0338	-.1232	-.0535	.0019	-.1062
EMOTION	.7425**	1.0000	-.1002	-.0451	-.2049**	-.0837	-.0689	-.1139
VOCABULARY	-.0153	-.1002	1.0000	.2016**	.5891**	.3543**	.4627**	.4985**
MATHABIL	-.0338	-.0451	.2016**	1.0000	.3940**	.3431**	.4133**	.3722**
KNOWLEDGE	-.1232	-.2049**	.5891**	.3940**	1.0000	.4595**	.6012**	.6781**
12THGPA	-.0535	-.0837	.3543**	.3431**	.4595**	1.0000	.7946**	.3570**
CUMGPA	.0019	-.0689	.4627**	.4133**	.6012**	.7946**	1.0000	.5346**

APTOTAL	-.1062	-.1139	.4985**	.3722**	.6781**	.3570**	.5346**	1.0000

* - Signif. LE .05   ** - Signif. LE .01   (2-tailed)   " . " is printed if a coefficient cannot be computed

Table S2. Supplementary table of intercorrlations among predictor and criterion variables. Number of participants with data are indicated for each pairwise correlation in parentheses.

	VERBAL	QUANT	FIGURAL	GENERAL	N	E	O	A	C	TIE
VERBAL	1.0000	.5899	.6114	.8349	-.2185	-.0892	.2278	.1265	-.0119	.2204
	(522)	(522)	(522)	(522)	(521)	(518)	(520)	(520)	(516)	(522)
	P= .	P= .000	P= .000	P= .000	P= .000	P= .043	P= .000	P= .004	P= .788	P= .000
QUANT	.5899	1.0000	.7541	.8612	-.1773	.0007	.0419	.1017	.0314	.1708
	(522)	(522)	(522)	(522)	(521)	(518)	(520)	(520)	(516)	(522)
	P= .000	P= .	P= .000	P= .000	P= .000	P= .987	P= .340	P= .020	P= .477	P= .000
FIGURAL	.6114	.7541	1.0000	.9246	-.1622	-.0368	.1300	.1336	.0271	.1609
	(522)	(522)	(522)	(522)	(521)	(518)	(520)	(520)	(516)	(522)
	P= .000	P= .000	P= .	P= .000	P= .000	P= .403	P= .003	P= .002	P= .539	P= .000
GENERAL	.8349	.8612	.9246	1.0000	-.2095	-.0513	.1606	.1406	.0174	.2078
	(522)	(522)	(522)	(522)	(521)	(518)	(520)	(520)	(516)	(522)
	P= .000	P= .000	P= .000	P= .	P= .000	P= .244	P= .000	P= .001	P= .694	P= .000
N	-.2185	-.1773	-.1622	-.2095	1.0000	-.2747	-.0193	-.3411	-.2891	-.1838
	(521)	(521)	(521)	(521)	(735)	(731)	(732)	(731)	(726)	(735)
	P= .000	P= .000	P= .000	P= .000	P= .	P= .000	P= .602	P= .000	P= .000	P= .000
E	-.0892	.0007	-.0368	-.0513	-.2747	1.0000	.0123	.2335	.2195	.0198
	(518)	(518)	(518)	(518)	(731)	(732)	(729)	(729)	(723)	(732)
	P= .043	P= .987	P= .403	P= .244	P= .000	P= .	P= .740	P= .000	P= .000	P= .594
O	.2278	.0419	.1300	.1606	-.0193	.0123	1.0000	-.0293	-.0321	.4142
	(520)	(520)	(520)	(520)	(732)	(729)	(735)	(731)	(723)	(735)
	P= .000	P= .340	P= .003	P= .000	P= .602	P= .740	P= .	P= .429	P= .389	P= .000
A	.1265	.1017	.1336	.1406	-.3411	.2335	-.0293	1.0000	.3121	.0449
	(520)	(520)	(520)	(520)	(731)	(729)	(731)	(734)	(723)	(734)
	P= .004	P= .020	P= .002	P= .001	P= .000	P= .000	P= .429	P= .	P= .000	P= .224
C	-.0119	.0314	.0271	.0174	-.2891	.2195	-.0321	.3121	1.0000	.2889

	(516)	(516)	(516)	(516)	(726)	(723)	(723)	(723)	(726)	(726)
	P= .788	P= .477	P= .539	P= .694	P= .000	P= .000	P= .389	P= .000	P= .	P= .000
TIE	.2204	.1708	.1609	.2078	-.1838	.0198	.4142	.0449	.2889	1.0000
	(522)	(522)	(522)	(522)	(735)	(732)	(735)	(734)	(726)	(738)
	P= .000	P= .000	P= .000	P= .000	P= .000	P= .594	P= .000	P= .224	P= .000	P= .

NACH	.1505	.1380	.1069	.1462	-.2678	.3166	.1271	.2007	.7725	.4054
	(522)	(522)	(522)	(522)	(735)	(732)	(735)	(734)	(726)	(738)
	P= .001	P= .002	P= .015	P= .001	P= .000	P= .000	P= .001	P= .000	P= .000	P= .000
SELFD	-.1121	-.0434	-.0582	-.0824	-.3245	.1641	-.0056	.2319	.7827	.2976
	(522)	(522)	(522)	(522)	(735)	(732)	(735)	(734)	(726)	(738)
	P= .010	P= .322	P= .184	P= .060	P= .000	P= .000	P= .879	P= .000	P= .000	P= .000
NUMPR	.0821	.3562	.2941	.2713	-.1365	.0781	-.0279	.0716	.2852	.3754
	(522)	(522)	(522)	(522)	(735)	(732)	(735)	(734)	(726)	(738)
	P= .061	P= .000	P= .000	P= .000	P= .000	P= .035	P= .450	P= .052	P= .000	P= .000
REALISTIC	.0122	.1150	.1720	.1202	-.0779	.0074	.0776	-.0554	.0780	.1728
	(522)	(522)	(522)	(522)	(735)	(732)	(734)	(733)	(726)	(737)
	P= .781	P= .009	P= .000	P= .006	P= .035	P= .842	P= .036	P= .134	P= .036	P= .000
INVEST	.1152	.1448	.1827	.1721	-.0213	-.0886	.3223	-.0084	.1535	.3944
	(521)	(521)	(521)	(521)	(734)	(731)	(734)	(733)	(725)	(737)
	P= .009	P= .001	P= .000	P= .000	P= .564	P= .017	P= .000	P= .820	P= .000	P= .000
ARTISTIC	.1608	.0087	.0977	.1109	.0854	.1507	.4786	-.0175	-.0148	.2337
	(522)	(522)	(522)	(522)	(735)	(732)	(735)	(734)	(726)	(738)
	P= .000	P= .843	P= .026	P= .011	P= .021	P= .000	P= .000	P= .636	P= .691	P= .000
SOCIAL	-.0961	-.0287	-.0221	-.0547	.0120	.4178	.1492	.0583	.1931	.2356
	(521)	(521)	(521)	(521)	(734)	(731)	(734)	(733)	(725)	(737)
	P= .028	P= .514	P= .614	P= .213	P= .745	P= .000	P= .000	P= .115	P= .000	P= .000
ENTERPRISE	-.1541	.0007	-.0392	-.0770	-.0582	.2828	.0633	-.0766	.2895	.2349
	(520)	(520)	(520)	(520)	(733)	(730)	(733)	(732)	(724)	(736)
	P= .000	P= .986	P= .372	P= .079	P= .115	P= .000	P= .087	P= .038	P= .000	P= .000
CONVENTION	-.1585	.1193	.1035	.0235	-.0220	.0666	-.0437	-.0643	.2663	.2193
	(522)	(522)	(522)	(522)	(734)	(731)	(734)	(733)	(725)	(737)
	P= .000	P= .006	P= .018	P= .593	P= .553	P= .072	P= .237	P= .082	P= .000	P= .000
VERBALSC	.2462	.0352	.0373	.1202	-.0860	.1252	.3884	-.0109	.2257	.4355
	(522)	(522)	(522)	(522)	(735)	(732)	(735)	(734)	(726)	(738)
	P= .000	P= .422	P= .396	P= .006	P= .020	P= .001	P= .000	P= .768	P= .000	P= .000

MATHSC	.1165	.3473	.3131	.2912	-.2063	.0783	-.0842	.0733	.2693	.2619
	(522)	(522)	(522)	(522)	(735)	(732)	(735)	(734)	(726)	(738)
	P= .008	P= .000	P= .000	P= .000	P= .000	P= .034	P= .022	P= .047	P= .000	P= .000

SCIENCSC	.2515	.2089	.2263	.2619	-.1982	-.0285	.2566	.0369	.2130	.4728
	(522)	(522)	(522)	(522)	(735)	(732)	(735)	(734)	(726)	(738)
	P= .000	P= .000	P= .000	P= .000	P= .000	P= .442	P= .000	P= .319	P= .000	P= .000
VERBALSE	.2841	.1897	.1834	.2478	-.1443	.1318	.2606	-.0137	.2108	.4218
	(521)	(521)	(521)	(521)	(734)	(731)	(734)	(733)	(725)	(737)
	P= .000	P= .000	P= .000	P= .000	P= .000	P= .000	P= .000	P= .712	P= .000	P= .000
MATHSE	.1838	.4470	.3807	.3758	-.2141	.0841	-.0530	.0593	.2324	.2599
	(521)	(521)	(521)	(521)	(734)	(731)	(734)	(733)	(725)	(737)
	P= .000	P= .000	P= .000	P= .000	P= .000	P= .023	P= .151	P= .109	P= .000	P= .000
SCIENCESE	.2900	.3025	.3255	.3500	-.1771	-.0460	.1214	.0390	.1276	.3098
	(521)	(521)	(521)	(521)	(734)	(731)	(734)	(733)	(725)	(737)
	P= .000	P= .000	P= .000	P= .000	P= .000	P= .214	P= .001	P= .292	P= .001	P= .000
GSE	.2574	.2708	.2521	.2932	-.1473	.0374	.1302	-.0038	.1972	.3146
	(521)	(521)	(521)	(521)	(734)	(731)	(734)	(733)	(725)	(737)
	P= .000	P= .000	P= .000	P= .000	P= .000	P= .313	P= .000	P= .919	P= .000	P= .000
DTL	.0950	.0792	.1059	.1091	-.1712	.1088	.3342	.0794	.5355	.5913
	(522)	(522)	(522)	(522)	(735)	(732)	(735)	(734)	(726)	(738)
	P= .030	P= .070	P= .015	P= .013	P= .000	P= .003	P= .000	P= .031	P= .000	P= .000
MASTERY	.0603	.1050	.0908	.0956	-.2256	.2785	.1475	.1640	.6927	.4228
	(520)	(520)	(520)	(520)	(733)	(730)	(733)	(732)	(725)	(736)
	P= .170	P= .017	P= .039	P= .029	P= .000	P= .000	P= .000	P= .000	P= .000	P= .000
OTHER	.0195	.0800	.0433	.0501	.1175	.0751	.0049	-.2098	.1745	.1580
	(521)	(521)	(521)	(521)	(734)	(731)	(734)	(733)	(725)	(737)
	P= .657	P= .068	P= .324	P= .254	P= .001	P= .042	P= .894	P= .000	P= .000	P= .000
COMPET	.0095	.1303	.0567	.0665	-.1247	.2465	.0021	-.2956	.0392	.0669
	(522)	(522)	(522)	(522)	(735)	(732)	(735)	(734)	(726)	(738)
	P= .828	P= .003	P= .196	P= .129	P= .001	P= .000	P= .955	P= .000	P= .291	P= .069
WORRY	-.0351	-.0213	-.0489	-.0430	.6336	-.1610	-.0288	-.1403	-.1391	-.0940
	(521)	(521)	(521)	(521)	(734)	(731)	(734)	(733)	(726)	(737)
	P= .424	P= .627	P= .265	P= .327	P= .000	P= .000	P= .436	P= .000	P= .000	P= .011

EMOTION	-.1256	-.1076	-.1223	-.1363	.6846	-.1432	-.0492	-.2236	-.2143	-.1836
	(522)	(522)	(522)	(522)	(735)	(732)	(735)	(734)	(726)	(738)
	P= .004	P= .014	P= .005	P= .002	P= .000	P= .000	P= .182	P= .000	P= .000	P= .000

VOCABULARY	.6447	.3490	.3688	.5253	-.0899	-.1033	.3392	.0420	.0589	.3301
	(219)	(219)	(219)	(219)	(253)	(253)	(253)	(252)	(253)	(254)
	P= .000	P= .000	P= .000	P= .000	P= .154	P= .101	P= .000	P= .507	P= .351	P= .000
MATHABIL	.3179	.3831	.4317	.4472	-.0592	.0175	.0908	.0475	.0765	.1755
	(210)	(210)	(210)	(210)	(239)	(239)	(239)	(238)	(238)	(240)
	P= .000	P= .000	P= .000	P= .000	P= .363	P= .787	P= .162	P= .465	P= .240	P= .006
KNOWLEDGE	.6183	.4960	.4198	.5786	-.1732	-.0877	.2695	.0747	.1130	.3142
	(246)	(246)	(246)	(246)	(283)	(283)	(283)	(283)	(281)	(284)
	P= .000	P= .000	P= .000	P= .000	P= .003	P= .141	P= .000	P= .210	P= .058	P= .000
12THGPA	.3752	.3171	.3266	.3868	-.0605	-.0018	.0248	.1600	.1836	.0727
	(522)	(522)	(522)	(522)	(735)	(732)	(735)	(734)	(726)	(738)
	P= .000	P= .000	P= .000	P= .000	P= .101	P= .961	P= .501	P= .000	P= .000	P= .048
CUMGPA	.4837	.4368	.4401	.5156	-.0698	-.0718	.0882	.1712	.2208	.1802
	(522)	(522)	(522)	(522)	(735)	(732)	(735)	(734)	(726)	(738)
	P= .000	P= .000	P= .000	P= .000	P= .059	P= .052	P= .017	P= .000	P= .000	P= .000
APTOTAL	.5309	.5479	.4709	.5783	-.1162	-.0744	.2363	.0394	.0867	.3039
	(522)	(522)	(522)	(522)	(735)	(732)	(735)	(734)	(726)	(738)
	P= .000	P= .000	P= .000	P= .000	P= .002	P= .044	P= .000	P= .287	P= .020	P= .000

	NACH	SELFD	NUMPR	REALISTIC	INVEST	ARTISTIC	SOCIAL	ENTERPRISE	CONVENTION	VSC
VERBAL	.1505	-.1121	.0821	.0122	.1152	.1608	-.0961	-.1541	-.1585	.2462
	(522)	(522)	(522)	(522)	(521)	(522)	(521)	(520)	(522)	(522)
	P= .001	P= .010	P= .061	P= .781	P= .009	P= .000	P= .028	P= .000	P= .000	P= .000
QUANT	.1380	-.0434	.3562	.1150	.1448	.0087	-.0287	.0007	.1193	.0352
	(522)	(522)	(522)	(522)	(521)	(522)	(521)	(520)	(522)	(522)
	P= .002	P= .322	P= .000	P= .009	P= .001	P= .843	P= .514	P= .986	P= .006	P= .422
FIGURAL	.1069	-.0582	.2941	.1720	.1827	.0977	-.0221	-.0392	.1035	.0373
	(522)	(522)	(522)	(522)	(521)	(522)	(521)	(520)	(522)	(522)
	P= .015	P= .184	P= .000	P= .000	P= .000	P= .026	P= .614	P= .372	P= .018	P= .396
GENERAL	.1462	-.0824	.2713	.1202	.1721	.1109	-.0547	-.0770	.0235	.1202
	(522)	(522)	(522)	(522)	(521)	(522)	(521)	(520)	(522)	(522)
	P= .001	P= .060	P= .000	P= .006	P= .000	P= .011	P= .213	P= .079	P= .593	P= .006
N	-.2678	-.3245	-.1365	-.0779	-.0213	.0854	.0120	-.0582	-.0220	-.0860
	(735)	(735)	(735)	(735)	(734)	(735)	(734)	(733)	(734)	(735)
	P= .000	P= .000	P= .000	P= .035	P= .564	P= .021	P= .745	P= .115	P= .553	P= .020
E	.3166	.1641	.0781	.0074	-.0886	.1507	.4178	.2828	.0666	.1252
	(732)	(732)	(732)	(732)	(731)	(732)	(731)	(730)	(731)	(732)
	P= .000	P= .000	P= .035	P= .842	P= .017	P= .000	P= .000	P= .000	P= .072	P= .001
O	.1271	-.0056	-.0279	.0776	.3223	.4786	.1492	.0633	-.0437	.3884
	(735)	(735)	(735)	(734)	(734)	(735)	(734)	(733)	(734)	(735)
	P= .001	P= .879	P= .450	P= .036	P= .000	P= .000	P= .000	P= .087	P= .237	P= .000
A	.2007	.2319	.0716	-.0554	-.0084	-.0175	.0583	-.0766	-.0643	-.0109
	(734)	(734)	(734)	(733)	(733)	(734)	(733)	(732)	(733)	(734)
	P= .000	P= .000	P= .052	P= .134	P= .820	P= .636	P= .115	P= .038	P= .082	P= .768
C	.7725	.7827	.2852	.0780	.1535	-.0148	.1931	.2895	.2663	.2257
	(726)	(726)	(726)	(726)	(725)	(726)	(725)	(724)	(725)	(726)
	P= .000	P= .000	P= .000	P= .036	P= .000	P= .691	P= .000	P= .000	P= .000	P= .000
TIE	.4054	.2976	.3754	.1728	.3944	.2337	.2356	.2349	.2193	.4355

	(738)	(738)	(738)	(737)	(737)	(738)	(737)	(736)	(737)	(738)
	P= .000	P= .000	P= .000	P= .000	P= .000	P= .000	P= .000	P= .000	P= .000	P= .000
NACH	1.0000	.6283	.3292	.1034	.2184	.1206	.2861	.3313	.2450	.2940
	(738)	(738)	(738)	(737)	(737)	(738)	(737)	(736)	(737)	(738)
	P= .	P= .000	P= .000	P= .005	P= .000	P= .001	P= .000	P= .000	P= .000	P= .000

SELFD	.6283	1.0000	.2226	.0529	.1381	-.0629	.1172	.2221	.1925	.1741
	(738)	(738)	(738)	(737)	(737)	(738)	(737)	(736)	(737)	(738)
	P= .000	P= .	P= .000	P= .151	P= .000	P= .088	P= .001	P= .000	P= .000	P= .000
NUMPR	.3292	.2226	1.0000	.2649	.2228	-.1110	.1255	.2009	.4887	-.0788
	(738)	(738)	(738)	(737)	(737)	(738)	(737)	(736)	(737)	(738)
	P= .000	P= .000	P= .	P= .000	P= .000	P= .003	P= .001	P= .000	P= .000	P= .032
REALISTIC	.1034	.0529	.2649	1.0000	.5132	.3214	.4003	.4104	.5482	.0078
	(737)	(737)	(737)	(737)	(736)	(737)	(736)	(735)	(736)	(737)
	P= .005	P= .151	P= .000	P= .	P= .000	P= .000	P= .000	P= .000	P= .000	P= .832
INVEST	.2184	.1381	.2228	.5132	1.0000	.2193	.2752	.2281	.2711	.1266
	(737)	(737)	(737)	(736)	(737)	(737)	(736)	(736)	(736)	(737)
	P= .000	P= .000	P= .000	P= .000	P= .	P= .000	P= .000	P= .000	P= .000	P= .001
ARTISTIC	.1206	-.0629	-.1110	.3214	.2193	1.0000	.4933	.3049	.0740	.4558
	(738)	(738)	(738)	(737)	(737)	(738)	(737)	(736)	(737)	(738)
	P= .001	P= .088	P= .003	P= .000	P= .000	P= .	P= .000	P= .000	P= .045	P= .000
SOCIAL	.2861	.1172	.1255	.4003	.2752	.4933	1.0000	.6491	.3607	.2831
	(737)	(737)	(737)	(736)	(736)	(737)	(737)	(735)	(736)	(737)
	P= .000	P= .001	P= .001	P= .000	P= .000	P= .000	P= .	P= .000	P= .000	P= .000
ENTERPRISE	.3313	.2221	.2009	.4104	.2281	.3049	.6491	1.0000	.6806	.2457
	(736)	(736)	(736)	(735)	(736)	(736)	(735)	(736)	(735)	(736)
	P= .000	P= .000	P= .000	P= .000	P= .000	P= .000	P= .000	P= .	P= .000	P= .000
CONVENTION	.2450	.1925	.4887	.5482	.2711	.0740	.3607	.6806	1.0000	.0250
	(737)	(737)	(737)	(736)	(736)	(737)	(736)	(735)	(737)	(737)
	P= .000	P= .000	P= .000	P= .000	P= .000	P= .045	P= .000	P= .000	P= .	P= .497
VERBALSC	.2940	.1741	-.0788	.0078	.1266	.4558	.2831	.2457	.0250	1.0000
	(738)	(738)	(738)	(737)	(737)	(738)	(737)	(736)	(737)	(738)
	P= .000	P= .000	P= .032	P= .832	P= .001	P= .000	P= .000	P= .000	P= .497	P= .
MATHSC	.3073	.2100	.7460	.2580	.1443	-.0980	.0814	.1886	.4277	-.0259
	(738)	(738)	(738)	(737)	(737)	(738)	(737)	(736)	(737)	(738)
	P= .000	P= .000	P= .000	P= .000	P= .000	P= .008	P= .027	P= .000	P= .000	P= .483

SCIENCSC	.2987	.1792	.2934	.2164	.5689	.0313	.0552	.1097	.1335	.2604
	(738)	(738)	(738)	(737)	(737)	(738)	(737)	(736)	(737)	(738)
	P= .000	P= .000	P= .000	P= .000	P= .000	P= .395	P= .134	P= .003	P= .000	P= .000

VERBALSE	.3140	.1965	.0247	.0691	.1884	.3646	.2262	.2135	.0418	.6442
	(737)	(737)	(737)	(736)	(736)	(737)	(736)	(735)	(736)	(737)
	P= .000	P= .000	P= .504	P= .061	P= .000	P= .000	P= .000	P= .000	P= .258	P= .000
MATHSE	.2955	.1830	.6305	.2405	.1620	-.0108	.0889	.1688	.3675	-.0350
	(737)	(737)	(737)	(736)	(736)	(737)	(736)	(735)	(736)	(737)
	P= .000	P= .000	P= .000	P= .000	P= .000	P= .769	P= .016	P= .000	P= .000	P= .342
SCIENCESE	.2302	.1167	.2369	.2175	.4751	.0363	.0136	.0163	.0603	.0995
	(737)	(737)	(737)	(736)	(736)	(737)	(736)	(735)	(736)	(737)
	P= .000	P= .002	P= .000	P= .000	P= .000	P= .325	P= .713	P= .659	P= .102	P= .007
GSE	.2875	.1453	.1882	.1552	.2160	.2010	.1209	.1766	.1397	.2953
	(737)	(737)	(737)	(736)	(736)	(737)	(736)	(735)	(736)	(737)
	P= .000	P= .000	P= .000	P= .000	P= .000	P= .000	P= .001	P= .000	P= .000	P= .000
DTL	.6355	.4702	.3897	.2021	.4467	.1645	.2356	.3014	.3061	.2867
	(738)	(738)	(738)	(737)	(737)	(738)	(737)	(736)	(737)	(738)
	P= .000	P= .000	P= .000	P= .000	P= .000	P= .000	P= .000	P= .000	P= .000	P= .000
MASTERY	.7927	.5659	.3643	.1362	.2673	.1170	.2775	.3375	.2858	.2203
	(736)	(736)	(736)	(735)	(735)	(736)	(735)	(734)	(735)	(736)
	P= .000	P= .000	P= .000	P= .000	P= .000	P= .001	P= .000	P= .000	P= .000	P= .000
OTHER	.2945	.0299	.1948	.1205	.1740	.0385	.1012	.2047	.1884	.0733
	(737)	(737)	(737)	(736)	(736)	(737)	(736)	(735)	(736)	(737)
	P= .000	P= .417	P= .000	P= .001	P= .000	P= .297	P= .006	P= .000	P= .000	P= .047
COMPET	.1684	.0120	.1285	.0737	.0503	-.0544	.0768	.1595	.0640	-.0136
	(738)	(738)	(738)	(737)	(737)	(738)	(737)	(736)	(737)	(738)
	P= .000	P= .746	P= .000	P= .046	P= .173	P= .140	P= .037	P= .000	P= .083	P= .713
WORRY	-.0860	-.2231	-.0506	-.0597	.0217	.0426	.0037	-.0261	.0139	-.0708
	(737)	(737)	(737)	(736)	(736)	(737)	(736)	(735)	(736)	(737)
	P= .019	P= .000	P= .170	P= .105	P= .557	P= .248	P= .920	P= .480	P= .706	P= .055
EMOTION	-.1741	-.2694	-.1193	-.0888	-.0557	.0641	.0403	-.0139	.0247	-.0837
	(738)	(738)	(738)	(737)	(737)	(738)	(737)	(736)	(737)	(738)
	P= .000	P= .000	P= .001	P= .016	P= .131	P= .082	P= .275	P= .706	P= .504	P= .023

VOCABULARY	.2023	-.0595	.1297	.0627	.2200	.2303	.0019	.0157	.0238	.2857
	(254)	(254)	(254)	(254)	(253)	(254)	(254)	(252)	(254)	(254)
	P= .001	P= .345	P= .039	P= .320	P= .000	P= .000	P= .976	P= .804	P= .705	P= .000

MATHABIL	.1651	.0089	.2641	.2497	.2292	.0482	.0314	-.0760	.0831	.0040
	(240)	(240)	(240)	(240)	(239)	(240)	(240)	(238)	(240)	(240)
	P= .010	P= .890	P= .000	P= .000	P= .000	P= .457	P= .628	P= .243	P= .199	P= .951
KNOWLEDGE	.2391	.0303	.2043	.0941	.2057	.1767	.0098	.0140	.0189	.2125
	(284)	(284)	(284)	(284)	(283)	(284)	(284)	(282)	(284)	(284)
	P= .000	P= .611	P= .001	P= .114	P= .000	P= .003	P= .869	P= .814	P= .751	P= .000
12THGPA	.1850	.0465	.1471	-.0054	.0655	.0448	.0212	-.0748	-.0187	.0384
	(738)	(738)	(738)	(737)	(737)	(738)	(737)	(736)	(737)	(738)
	P= .000	P= .207	P= .000	P= .884	P= .076	P= .225	P= .566	P= .043	P= .612	P= .298
CUMGPA	.2896	.0678	.2359	-.0218	.1118	.0435	-.0081	-.0833	-.0014	.0812
	(738)	(738)	(738)	(737)	(737)	(738)	(737)	(736)	(737)	(738)
	P= .000	P= .065	P= .000	P= .555	P= .002	P= .238	P= .825	P= .024	P= .970	P= .027
APTOTAL	.2295	-.0263	.2529	.0958	.1910	.1269	.0174	.0369	.0650	.1515
	(738)	(738)	(738)	(737)	(737)	(738)	(737)	(736)	(737)	(738)
	P= .000	P= .476	P= .000	P= .009	P= .000	P= .001	P= .638	P= .317	P= .078	P= .000

	MATHSC	SCIENCSC	VERBALSE	MATHSE	SCIENCESE	GSE	DTL	MASTERY	OTHER	COMPET
VERBAL	.1165	.2515	.2841	.1838	.2900	.2574	.0950	.0603	.0195	.0095
	(522)	(522)	(521)	(521)	(521)	(521)	(522)	(520)	(521)	(522)
	P= .008	P= .000	P= .000	P= .000	P= .000	P= .000	P= .030	P= .170	P= .657	P= .828
QUANT	.3473	.2089	.1897	.4470	.3025	.2708	.0792	.1050	.0800	.1303
	(522)	(522)	(521)	(521)	(521)	(521)	(522)	(520)	(521)	(522)
	P= .000	P= .000	P= .000	P= .000	P= .000	P= .000	P= .070	P= .017	P= .068	P= .003
FIGURAL	.3131	.2263	.1834	.3807	.3255	.2521	.1059	.0908	.0433	.0567
	(522)	(522)	(521)	(521)	(521)	(521)	(522)	(520)	(521)	(522)
	P= .000	P= .000	P= .000	P= .000	P= .000	P= .000	P= .015	P= .039	P= .324	P= .196
GENERAL	.2912	.2619	.2478	.3758	.3500	.2932	.1091	.0956	.0501	.0665
	(522)	(522)	(521)	(521)	(521)	(521)	(522)	(520)	(521)	(522)
	P= .000	P= .000	P= .000	P= .000	P= .000	P= .000	P= .013	P= .029	P= .254	P= .129
N	-.2063	-.1982	-.1443	-.2141	-.1771	-.1473	-.1712	-.2256	.1175	-.1247
	(735)	(735)	(734)	(734)	(734)	(734)	(735)	(733)	(734)	(735)
	P= .000	P= .000	P= .000	P= .000	P= .000	P= .000	P= .000	P= .000	P= .001	P= .001
E	.0783	-.0285	.1318	.0841	-.0460	.0374	.1088	.2785	.0751	.2465
	(732)	(732)	(731)	(731)	(731)	(731)	(732)	(730)	(731)	(732)
	P= .034	P= .442	P= .000	P= .023	P= .214	P= .313	P= .003	P= .000	P= .042	P= .000
O	-.0842	.2566	.2606	-.0530	.1214	.1302	.3342	.1475	.0049	.0021
	(735)	(735)	(734)	(734)	(734)	(734)	(735)	(733)	(734)	(735)
	P= .022	P= .000	P= .000	P= .151	P= .001	P= .000	P= .000	P= .000	P= .894	P= .955
A	.0733	.0369	-.0137	.0593	.0390	-.0038	.0794	.1640	-.2098	-.2956
	(734)	(734)	(733)	(733)	(733)	(733)	(734)	(732)	(733)	(734)
	P= .047	P= .319	P= .712	P= .109	P= .292	P= .919	P= .031	P= .000	P= .000	P= .000
C	.2693	.2130	.2108	.2324	.1276	.1972	.5355	.6927	.1745	.0392
	(726)	(726)	(725)	(725)	(725)	(725)	(726)	(725)	(725)	(726)
	P= .000	P= .000	P= .000	P= .000	P= .001	P= .000	P= .000	P= .000	P= .000	P= .291
TIE	.2619	.4728	.4218	.2599	.3098	.3146	.5913	.4228	.1580	.0669

	(738)	(738)	(737)	(737)	(737)	(737)	(738)	(736)	(737)	(738)
	P= .000	P= .000	P= .000	P= .000	P= .000	P= .000	P= .000	P= .000	P= .000	P= .069
NACH	.3073	.2987	.3140	.2955	.2302	.2875	.6355	.7927	.2945	.1684
	(738)	(738)	(737)	(737)	(737)	(737)	(738)	(736)	(737)	(738)
	P= .000	P= .000	P= .000	P= .000	P= .000	P= .000	P= .000	P= .000	P= .000	P= .000

SELFD	.2100	.1792	.1965	.1830	.1167	.1453	.4702	.5659	.0299	.0120
	(738)	(738)	(737)	(737)	(737)	(737)	(738)	(736)	(737)	(738)
	P= .000	P= .000	P= .000	P= .000	P= .002	P= .000	P= .000	P= .000	P= .417	P= .746
NUMPR	.7460	.2934	.0247	.6305	.2369	.1882	.3897	.3643	.1948	.1285
	(738)	(738)	(737)	(737)	(737)	(737)	(738)	(736)	(737)	(738)
	P= .000	P= .000	P= .504	P= .000	P= .000	P= .000	P= .000	P= .000	P= .000	P= .000
REALISTIC	.2580	.2164	.0691	.2405	.2175	.1552	.2021	.1362	.1205	.0737
	(737)	(737)	(736)	(736)	(736)	(736)	(737)	(735)	(736)	(737)
	P= .000	P= .000	P= .061	P= .000	P= .000	P= .000	P= .000	P= .000	P= .001	P= .046
INVEST	.1443	.5689	.1884	.1620	.4751	.2160	.4467	.2673	.1740	.0503
	(737)	(737)	(736)	(736)	(736)	(736)	(737)	(735)	(736)	(737)
	P= .000	P= .000	P= .000	P= .000	P= .000	P= .000	P= .000	P= .000	P= .000	P= .173
ARTISTIC	-.0980	.0313	.3646	-.0108	.0363	.2010	.1645	.1170	.0385	-.0544
	(738)	(738)	(737)	(737)	(737)	(737)	(738)	(736)	(737)	(738)
	P= .008	P= .395	P= .000	P= .769	P= .325	P= .000	P= .000	P= .001	P= .297	P= .140
SOCIAL	.0814	.0552	.2262	.0889	.0136	.1209	.2356	.2775	.1012	.0768
	(737)	(737)	(736)	(736)	(736)	(736)	(737)	(735)	(736)	(737)
	P= .027	P= .134	P= .000	P= .016	P= .713	P= .001	P= .000	P= .000	P= .006	P= .037
ENTERPRISE	.1886	.1097	.2135	.1688	.0163	.1766	.3014	.3375	.2047	.1595
	(736)	(736)	(735)	(735)	(735)	(735)	(736)	(734)	(735)	(736)
	P= .000	P= .003	P= .000	P= .000	P= .659	P= .000	P= .000	P= .000	P= .000	P= .000
CONVENTION	.4277	.1335	.0418	.3675	.0603	.1397	.3061	.2858	.1884	.0640
	(737)	(737)	(736)	(736)	(736)	(736)	(737)	(735)	(736)	(737)
	P= .000	P= .000	P= .258	P= .000	P= .102	P= .000	P= .000	P= .000	P= .000	P= .083
VERBALSC	-.0259	.2604	.6442	-.0350	.0995	.2953	.2867	.2203	.0733	-.0136
	(738)	(738)	(737)	(737)	(737)	(737)	(738)	(736)	(737)	(738)
	P= .483	P= .000	P= .000	P= .342	P= .007	P= .000	P= .000	P= .000	P= .047	P= .713
MATHSC	1.0000	.3490	.0549	.7654	.2447	.2479	.3118	.2755	.1423	.0974
	(738)	(738)	(737)	(737)	(737)	(737)	(738)	(736)	(737)	(738)
	P= .	P= .000	P= .137	P= .000	P= .000	P= .000	P= .000	P= .000	P= .000	P= .008

SCIENCSC	.3490	1.0000	.2828	.2914	.7022	.3280	.4528	.2542	.1654	.0613
	(738)	(738)	(737)	(737)	(737)	(737)	(738)	(736)	(737)	(738)
	P= .000	P= .	P= .000	P= .000	P= .000	P= .000	P= .000	P= .000	P= .000	P= .096

VERBALSE	.0549	.2828	1.0000	.3284	.4019	.6492	.3210	.2503	.0776	.0203
	(737)	(737)	(737)	(737)	(737)	(737)	(737)	(735)	(736)	(737)
	P= .137	P= .000	P= .	P= .000	P= .000	P= .000	P= .000	P= .000	P= .035	P= .582
MATHSE	.7654	.2914	.3284	1.0000	.4425	.5135	.3051	.2834	.1717	.1251
	(737)	(737)	(737)	(737)	(737)	(737)	(737)	(735)	(736)	(737)
	P= .000	P= .000	P= .000	P= .	P= .000	P= .000	P= .000	P= .000	P= .000	P= .001
SCIENCESE	.2447	.7022	.4019	.4425	1.0000	.4824	.3152	.1829	.1337	.0575
	(737)	(737)	(737)	(737)	(737)	(737)	(737)	(735)	(736)	(737)
	P= .000	P= .000	P= .000	P= .000	P= .	P= .000	P= .000	P= .000	P= .000	P= .119
GSE	.2479	.3280	.6492	.5135	.4824	1.0000	.3146	.2314	.1525	.0935
	(737)	(737)	(737)	(737)	(737)	(737)	(737)	(735)	(736)	(737)
	P= .000	P= .000	P= .000	P= .000	P= .000	P= .	P= .000	P= .000	P= .000	P= .011
DTL	.3118	.4528	.3210	.3051	.3152	.3146	1.0000	.7257	.2922	.0858
	(738)	(738)	(737)	(737)	(737)	(737)	(738)	(736)	(737)	(738)
	P= .000	P= .000	P= .000	P= .000	P= .000	P= .000	P= .	P= .000	P= .000	P= .020
MASTERY	.2755	.2542	.2503	.2834	.1829	.2314	.7257	1.0000	.3522	.2099
	(736)	(736)	(735)	(735)	(735)	(735)	(736)	(736)	(735)	(736)
	P= .000	P= .000	P= .000	P= .000	P= .000	P= .000	P= .000	P= .	P= .000	P= .000
OTHER	.1423	.1654	.0776	.1717	.1337	.1525	.2922	.3522	1.0000	.5737
	(737)	(737)	(736)	(736)	(736)	(736)	(737)	(735)	(737)	(737)
	P= .000	P= .000	P= .035	P= .000	P= .000	P= .000	P= .000	P= .000	P= .	P= .000
COMPET	.0974	.0613	.0203	.1251	.0575	.0935	.0858	.2099	.5737	1.0000
	(738)	(738)	(737)	(737)	(737)	(737)	(738)	(736)	(737)	(738)
	P= .008	P= .096	P= .582	P= .001	P= .119	P= .011	P= .020	P= .000	P= .000	P= .
WORRY	-.1489	-.1390	-.0936	-.1424	-.1451	-.1151	-.0795	-.0455	.3480	.0179
	(737)	(737)	(736)	(736)	(736)	(736)	(737)	(735)	(736)	(737)
	P= .000	P= .000	P= .011	P= .000	P= .000	P= .002	P= .031	P= .218	P= .000	P= .628
EMOTION	-.1954	-.2193	-.1363	-.1944	-.2144	-.1705	-.1550	-.0868	.1951	-.0758
	(738)	(738)	(737)	(737)	(737)	(737)	(738)	(736)	(737)	(738)
	P= .000	P= .000	P= .000	P= .000	P= .000	P= .000	P= .000	P= .019	P= .000	P= .040

VOCABULARY	.0979	.2330	.3807	.1813	.2546	.2154	.2157	.0917	.0720	.0815
	(254)	(254)	(253)	(253)	(253)	(253)	(254)	(254)	(254)	(254)
	P= .120	P= .000	P= .000	P= .004	P= .000	P= .001	P= .001	P= .145	P= .253	P= .195

MATHABIL	.2945	.2287	.0340	.2911	.2850	.1218	.1642	.1778	.2107	.1947
	(240)	(240)	(239)	(239)	(239)	(239)	(240)	(240)	(240)	(240)
	P= .000	P= .000	P= .601	P= .000	P= .000	P= .060	P= .011	P= .006	P= .001	P= .002
KNOWLEDGE	.2305	.3280	.3567	.3174	.3590	.3452	.3042	.1580	.1204	.0794
	(284)	(284)	(283)	(283)	(283)	(283)	(284)	(284)	(284)	(284)
	P= .000	P= .000	P= .000	P= .000	P= .000	P= .000	P= .000	P= .008	P= .043	P= .182
12THGPA	.2332	.1416	.0589	.2165	.1501	.0998	.0560	.1034	.1019	.0067
	(738)	(738)	(737)	(737)	(737)	(737)	(738)	(736)	(737)	(738)
	P= .000	P= .000	P= .110	P= .000	P= .000	P= .007	P= .128	P= .005	P= .006	P= .855
CUMGPA	.3160	.2218	.1291	.3220	.2500	.1892	.1376	.2020	.1683	.0120
	(738)	(738)	(737)	(737)	(737)	(737)	(738)	(736)	(737)	(738)
	P= .000	P= .000	P= .000	P= .000	P= .000	P= .000	P= .000	P= .000	P= .000	P= .745
APTOTAL	.2896	.2881	.2459	.3349	.3168	.3179	.2265	.1448	.1616	.0638
	(738)	(738)	(737)	(737)	(737)	(737)	(738)	(736)	(737)	(738)
	P= .000	P= .000	P= .000	P= .000	P= .000	P= .000	P= .000	P= .000	P= .000	P= .083

	WORRY	EMOTION	VOCABULARY	MATHABIL	KNOWLEDGE	12THGPA	CUMGPA	APTOT
VERBAL	-.0351	-.1256	.6447	.3179	.6183	.3752	.4837	.5309
	(521)	(522)	(219)	(210)	(246)	(522)	(522)	(522)
	P= .424	P= .004	P= .000	P= .000	P= .000	P= .000	P= .000	P= .000
QUANT	-.0213	-.1076	.3490	.3831	.4960	.3171	.4368	.5479
	(521)	(522)	(219)	(210)	(246)	(522)	(522)	(522)
	P= .627	P= .014	P= .000	P= .000	P= .000	P= .000	P= .000	P= .000
FIGURAL	-.0489	-.1223	.3688	.4317	.4198	.3266	.4401	.4709
	(521)	(522)	(219)	(210)	(246)	(522)	(522)	(522)
	P= .265	P= .005	P= .000	P= .000	P= .000	P= .000	P= .000	P= .000
GENERAL	-.0430	-.1363	.5253	.4472	.5786	.3868	.5156	.5783
	(521)	(522)	(219)	(210)	(246)	(522)	(522)	(522)
	P= .327	P= .002	P= .000	P= .000	P= .000	P= .000	P= .000	P= .000
N	.6336	.6846	-.0899	-.0592	-.1732	-.0605	-.0698	-.1162
	(734)	(735)	(253)	(239)	(283)	(735)	(735)	(735)
	P= .000	P= .000	P= .154	P= .363	P= .003	P= .101	P= .059	P= .002
E	-.1610	-.1432	-.1033	.0175	-.0877	-.0018	-.0718	-.0744
	(731)	(732)	(253)	(239)	(283)	(732)	(732)	(732)
	P= .000	P= .000	P= .101	P= .787	P= .141	P= .961	P= .052	P= .044
O	-.0288	-.0492	.3392	.0908	.2695	.0248	.0882	.2363
	(734)	(735)	(253)	(239)	(283)	(735)	(735)	(735)
	P= .436	P= .182	P= .000	P= .162	P= .000	P= .501	P= .017	P= .000
A	-.1403	-.2236	.0420	.0475	.0747	.1600	.1712	.0394
	(733)	(734)	(252)	(238)	(283)	(734)	(734)	(734)
	P= .000	P= .000	P= .507	P= .465	P= .210	P= .000	P= .000	P= .287
C	-.1391	-.2143	.0589	.0765	.1130	.1836	.2208	.0867
	(726)	(726)	(253)	(238)	(281)	(726)	(726)	(726)
	P= .000	P= .000	P= .351	P= .240	P= .058	P= .000	P= .000	P= .020
TIE	-.0940	-.1836	.3301	.1755	.3142	.0727	.1802	.3039

	(737)	(738)	(254)	(240)	(284)	(738)	(738)	(738)
	P= .011	P= .000	P= .000	P= .006	P= .000	P= .048	P= .000	P= .000
NACH	-.0860	-.1741	.2023	.1651	.2391	.1850	.2896	.2295
	(737)	(738)	(254)	(240)	(284)	(738)	(738)	(738)
	P= .019	P= .000	P= .001	P= .010	P= .000	P= .000	P= .000	P= .000

SELFD	-.2231	-.2694	-.0595	.0089	.0303	.0465	.0678	-.0263
	(737)	(738)	(254)	(240)	(284)	(738)	(738)	(738)
	P= .000	P= .000	P= .345	P= .890	P= .611	P= .207	P= .065	P= .476
NUMPR	-.0506	-.1193	.1297	.2641	.2043	.1471	.2359	.2529
	(737)	(738)	(254)	(240)	(284)	(738)	(738)	(738)
	P= .170	P= .001	P= .039	P= .000	P= .001	P= .000	P= .000	P= .000
REALISTIC	-.0597	-.0888	.0627	.2497	.0941	-.0054	-.0218	.0958
	(736)	(737)	(254)	(240)	(284)	(737)	(737)	(737)
	P= .105	P= .016	P= .320	P= .000	P= .114	P= .884	P= .555	P= .009
INVEST	.0217	-.0557	.2200	.2292	.2057	.0655	.1118	.1910
	(736)	(737)	(253)	(239)	(283)	(737)	(737)	(737)
	P= .557	P= .131	P= .000	P= .000	P= .000	P= .076	P= .002	P= .000
ARTISTIC	.0426	.0641	.2303	.0482	.1767	.0448	.0435	.1269
	(737)	(738)	(254)	(240)	(284)	(738)	(738)	(738)
	P= .248	P= .082	P= .000	P= .457	P= .003	P= .225	P= .238	P= .001
SOCIAL	.0037	.0403	.0019	.0314	.0098	.0212	-.0081	.0174
	(736)	(737)	(254)	(240)	(284)	(737)	(737)	(737)
	P= .920	P= .275	P= .976	P= .628	P= .869	P= .566	P= .825	P= .638
ENTERPRISE	-.0261	-.0139	.0157	-.0760	.0140	-.0748	-.0833	.0369
	(735)	(736)	(252)	(238)	(282)	(736)	(736)	(736)
	P= .480	P= .706	P= .804	P= .243	P= .814	P= .043	P= .024	P= .317
CONVENTION	.0139	.0247	.0238	.0831	.0189	-.0187	-.0014	.0650
	(736)	(737)	(254)	(240)	(284)	(737)	(737)	(737)
	P= .706	P= .504	P= .705	P= .199	P= .751	P= .612	P= .970	P= .078
VERBALSC	-.0708	-.0837	.2857	.0040	.2125	.0384	.0812	.1515
	(737)	(738)	(254)	(240)	(284)	(738)	(738)	(738)
	P= .055	P= .023	P= .000	P= .951	P= .000	P= .298	P= .027	P= .000
MATHSC	-.1489	-.1954	.0979	.2945	.2305	.2332	.3160	.2896
	(737)	(738)	(254)	(240)	(284)	(738)	(738)	(738)
	P= .000	P= .000	P= .120	P= .000	P= .000	P= .000	P= .000	P= .000

SCIENCSC	-.1390	-.2193	.2330	.2287	.3280	.1416	.2218	.2881
	(737)	(738)	(254)	(240)	(284)	(738)	(738)	(738)
	P= .000	P= .000	P= .000	P= .000	P= .000	P= .000	P= .000	P= .000

VERBALSE	-.0936	-.1363	.3807	.0340	.3567	.0589	.1291	.2459
	(736)	(737)	(253)	(239)	(283)	(737)	(737)	(737)
	P= .011	P= .000	P= .000	P= .601	P= .000	P= .110	P= .000	P= .000
MATHSE	-.1424	-.1944	.1813	.2911	.3174	.2165	.3220	.3349
	(736)	(737)	(253)	(239)	(283)	(737)	(737)	(737)
	P= .000	P= .000	P= .004	P= .000	P= .000	P= .000	P= .000	P= .000
SCIENCESE	-.1451	-.2144	.2546	.2850	.3590	.1501	.2500	.3168
	(736)	(737)	(253)	(239)	(283)	(737)	(737)	(737)
	P= .000	P= .000	P= .000	P= .000	P= .000	P= .000	P= .000	P= .000
GSE	-.1151	-.1705	.2154	.1218	.3452	.0998	.1892	.3179
	(736)	(737)	(253)	(239)	(283)	(737)	(737)	(737)
	P= .002	P= .000	P= .001	P= .060	P= .000	P= .007	P= .000	P= .000
DTL	-.0795	-.1550	.2157	.1642	.3042	.0560	.1376	.2265
	(737)	(738)	(254)	(240)	(284)	(738)	(738)	(738)
	P= .031	P= .000	P= .001	P= .011	P= .000	P= .128	P= .000	P= .000
MASTERY	-.0455	-.0868	.0917	.1778	.1580	.1034	.2020	.1448
	(735)	(736)	(254)	(240)	(284)	(736)	(736)	(736)
	P= .218	P= .019	P= .145	P= .006	P= .008	P= .005	P= .000	P= .000
OTHER	.3480	.1951	.0720	.2107	.1204	.1019	.1683	.1616
	(736)	(737)	(254)	(240)	(284)	(737)	(737)	(737)
	P= .000	P= .000	P= .253	P= .001	P= .043	P= .006	P= .000	P= .000
COMPET	.0179	-.0758	.0815	.1947	.0794	.0067	.0120	.0638
	(737)	(738)	(254)	(240)	(284)	(738)	(738)	(738)
	P= .628	P= .040	P= .195	P= .002	P= .182	P= .855	P= .745	P= .083
WORRY	1.0000	.7316	.0178	.0233	-.0559	.0717	.0483	.0068
	(737)	(737)	(254)	(240)	(284)	(737)	(737)	(737)
	P= .	P= .000	P= .777	P= .719	P= .348	P= .052	P= .190	P= .853
EMOTION	.7316	1.0000	-.0886	-.0019	-.1300	-.0305	-.0602	-.0936
	(737)	(738)	(254)	(240)	(284)	(738)	(738)	(738)
	P= .000	P= .	P= .159	P= .976	P= .029	P= .409	P= .102	P= .011

VOCABULARY	.0178	-.0886	1.0000	.2434	.5792	.3301	.4540	.4870
	(254)	(254)	(254)	(225)	(227)	(254)	(254)	(254)
	P= .777	P= .159	P= .	P= .000	P= .000	P= .000	P= .000	P= .000

MATHABIL	.0233	-.0019	.2434	1.0000	.4061	.3159	.3985	.3859
	(240)	(240)	(225)	(240)	(220)	(240)	(240)	(240)
	P= .719	P= .976	P= .000	P= .	P= .000	P= .000	P= .000	P= .000
KNOWLEDGE	-.0559	-.1300	.5792	.4061	1.0000	.3854	.5778	.6718
	(284)	(284)	(227)	(220)	(284)	(284)	(284)	(284)
	P= .348	P= .029	P= .000	P= .000	P= .	P= .000	P= .000	P= .000
12THGPA	.0717	-.0305	.3301	.3159	.3854	1.0000	.8286	.3401
	(737)	(738)	(254)	(240)	(284)	(738)	(738)	(738)
	P= .052	P= .409	P= .000	P= .000	P= .000	P= .	P= .000	P= .000
CUMGPA	.0483	-.0602	.4540	.3985	.5778	.8286	1.0000	.5431
	(737)	(738)	(254)	(240)	(284)	(738)	(738)	(738)
	P= .190	P= .102	P= .000	P= .000	P= .000	P= .000	P= .	P= .000
APTOTAL	.0068	-.0936	.4870	.3859	.6718	.3401	.5431	1.0000
	(737)	(738)	(254)	(240)	(284)	(738)	(738)	(738)
	P= .853	P= .011	P= .000	P= .000	P= .000	P= .000	P= .000	P= .

Table 3. Abbreviations in Table 1 and Table 2.

VERBAL = CogAT composite of verbal abilities tests
QUANT = CogAT composite of quantitative abilities tests
FIGURAL = CogAT composite of figural abilities tests
GENERAL = CogAT composite of all tests (part-whole correlations with ability tests)
N = NEO-FFI Neuroticism
E = NEO-FFI Extroversion
O = NEO-FFI Openness to Experience
A = NEO-FFI Agreeableness
C = NEO-FFI Conscientiousness
TIE = Goff & Ackerman Typical Intellectual Engagement - short form
NACH = International Personality Item Pool Need for Achievement
SELFD = International Personality Item Pool Self-Discipline
NUMPR = short version of Viswanathan (1993) Preference for Numerical Information scale
REALISTIC = UNIACT Realistic Interest Theme
INVEST = UNIACT Investigative Interest Theme
ARTISTIC = UNIACT Artitistic Interest Theme
SOCIAL = UNIACT Social Interest Theme
ENTERPRISE = UNIACT Enterprising Interest Theme
CONVENTION = UNIACT Conventional Interest Theme
VERBALSC = Verbal Self-Concept (locally developed)
MATHSC = Math Self-Concept (locally developed)
SCIENCSC = Science Self-Concept (locally developed)
VERBALSE = Verbal Self-Ratings of Ability (locally developed)
MATHSE = Math Self-Ratings of Ability (locally developed)
SCIENCESE = Science Self-Ratings of Ability (locally developed)
GSE = General Intelligence Self-Ratings of Ability (locally developed)
DTL = Motivational Trait Questionnaire Short Form Desire to Learn
MASTERY = Motivational Trait Questionnaire Short Form Mastery
OTHER = Motivational Trait Questionnaire Short Form Other-Oriented Goals
COMPET = Motivational Trait Questionnaire Short Form Competitiveness
WORRY = Motivational Trait Questionnaire Short Form Worry in Achievement Contexts
EMOTION = Motivational Trait Questionnaire Short Form Emotion in Achievement Contexts
VOCABULARY = ETS Extended Range Vocabulary Test
MATHABIL = Math Ability (see Ackerman & Kanfer, 1993)
KNOWLEDGE = Composite of six knowledge tests (Biology, Chemistry, U.S. Government, U.S. History, U.S. Literature, and Western Civilization) - (Rolfhus & Ackerman, 1999)
12THGPA = Grade Point Average (12th Grade Only)

CUMGPA = Cumulative Grade Point Average (9th-12th Grade)
APTOTAL = Total Number of Advanced Placement courses completed
